# SMYD2 glutathionylation contributes to degradation of sarcomeric proteins

**DOI:** 10.1038/s41467-018-06786-x

**Published:** 2018-10-18

**Authors:** Dhanushka N. P. Munkanatta Godage, Garrett C. VanHecke, Kusal T. G. Samarasinghe, Han-Zhong Feng, Mark Hiske, Joshua Holcomb, Zhe Yang, Jian-Ping Jin, Charles S. Chung, Young-Hoon Ahn

**Affiliations:** 1 Department of Chemistry, Wayne State University, Detroit, MI 48202 USA; 2 Department of Physiology, Wayne State University School of Medicine, Detroit, MI 48201 USA; 3 Department of Biochemistry and Molecular Biology, Wayne State University School of Medicine, Detroit, MI 48201 USA

## Abstract

Reactive oxygen species (ROS) contribute to the etiology of multiple muscle-related diseases. There is emerging evidence that cellular stress can lead to destabilization of sarcomeres, the contractile unit of muscle. However, it is incompletely understood how cellular stress induces structural destabilization of sarcomeres. Here we report that glutathionylation of SMYD2 contributes to a loss of myofibril integrity and degradation of sarcomeric proteins mediated by MMP-2 and calpain 1. We used a clickable glutathione approach in a cardiomyocyte cell line and found selective glutathionylation of SMYD2 at Cys13. Biochemical analysis demonstrated that SMYD2 upon oxidation or glutathionylation at Cys13 loses its interaction with Hsp90 and N2A, a domain of titin. Upon dissociation from SMYD2, N2A or titin is degraded by activated MMP-2, suggesting a protective role of SMYD2 in sarcomere stability. Taken together, our results support that SMYD2 glutathionylation is a novel molecular mechanism by which ROS contribute to sarcomere destabilization.

## Abstract (web summary)

Ischemic reperfusion or nutrient deprivation that produces reactive oxygen species can lead to a loss of muscle contractile function. Here the authors show that glutathionylation of the lysine methyltransferase SMYD2 contributes to degradation or disassembly of sarcomeres.

## Introduction

Reactive oxygen species (ROS) play an essential role in redox signaling, but also cause detrimental effects under oxidative stress, especially in cardiac and skeletal muscle.^1–3^ For example, low levels of ROS are produced in muscle, regulating intracellular calcium release and contractile force.^4,5^ Hydrogen peroxide (H_2_O_2_) derived from the endothelium and myocardial mitochondria serves as a vasodilator.^6–8^ In contrast, high levels of ROS induce abnormal calcium regulation, contractile dysfunction, and hypertrophy.^1^ Indeed, elevated levels of ROS are associated with muscle-related pathological conditions, including myocardial ischemia-reperfusion injury,^9^ heart failure,^10^ and muscular dystrophy.^11^

The basic contractile unit of striated muscle is a sarcomere. Sarcomeres are composed of multiple sarcomeric proteins, including actin, myosin, and titin, that are assembled in a highly organized structure. However, sarcomeric proteins are vulnerable to accelerated degradation under certain conditions, such as ischemic-reperfusion injury or nutrient deprivation that involves production of mitochondrial ROS.^12–14^ For example, ischemia-reperfusion injury leads to an increased activity of several proteases, including MMP-2 and calpain 1/3.^12–14^ It is well-known that MMP-2 is induced and activated under oxidative stress.^12^ Activated MMP-2 and calpains are involved in degradation of several sarcomeric proteins, including titin, α-actinin, troponin, and myosin-light chains.^15^ In particular, titin serves as an integral part of a stress-sensing network.^16^ The elastic region of titin binds with chaperones, proteases, and signaling complexes of which interactions are altered in response to the mechanical and chemical stress, ultimately causing muscle degradation, remodeling, or adaptation to stress.^16^ Despite these analyses, molecular mechanisms by which ROS induce structural destabilization or degradation of sarcomeres are incompletely understood.

A direct consequence of ROS in the sarcomere is oxidative protein modifications.^17,18^ In particular, glutathionylation is one of the major oxidative protein modifications in response to ROS.^19^ We recently developed a clickable glutathione approach to identify protein glutathionylation.^20,21^ In this approach, azido-glutathione (γGlu-Cys-azido-Ala, N_3_-GSH) is in situ biosynthesized in cells expressing a glutathione synthetase mutant (GS M4) that efficiently catalyzes an incorporation of azido-Ala in place of Gly to glutathione (γGlu-Cys-Gly, GSH).^20^ With this approach, we recently demonstrated that glucose depletion or treatment of mitochondrial electron transport chain blockers strongly induces global protein glutathionylation.^22^ Further mass spectrometric and biochemical analysis identified multiple glutathionylated proteins, including SET and MYND domain-containing protein 2 (SMYD2).^22^

SMYD2 is one of SET and MYND-containing lysine methyltransferases (SMYD).^23^ There are five members of the SMYD family (SMYD1-5). SMYD2 is mostly cytoplasmic where it catalyzes mono-methylation of p53,^24^ retinoblastoma protein (Rb),^25^ estrogen receptor α (ERα),^26^ and heat shock protein 90 (Hsp90).^27^ Recent proteomic analysis found numerous substrate proteins of SMYD2, suggesting its roles in diverse cellular processes.^28^ Importantly, both SMYD1 and SMYD2 are abundant in cardiac and skeletal muscle, playing an important role in myofibril assembly.^29^ At a molecular level, SMYD2 forms a complex with Hsp90.^30^ This complex binds to the N2A domain of titin at the I-band of sarcomere.^30^ Recently, it was found that zebrafish with knockdown of SMYD2 (a or a/b isoforms) forms disorganized I-bands and Z-disks in the sarcomere of muscle,^31^ suggesting its role in sarcomere stabilization. In this report, we show that SMYD2 can be selectively glutathionylated at Cys13, and SMYD2 Cys13 glutathionylation serves as a molecular event that contributes to degradation of sarcomeric proteins in response to ROS.

## Results

### SMYD2 is susceptible to glutathionylation in response to ROS

**Fig. 1:**
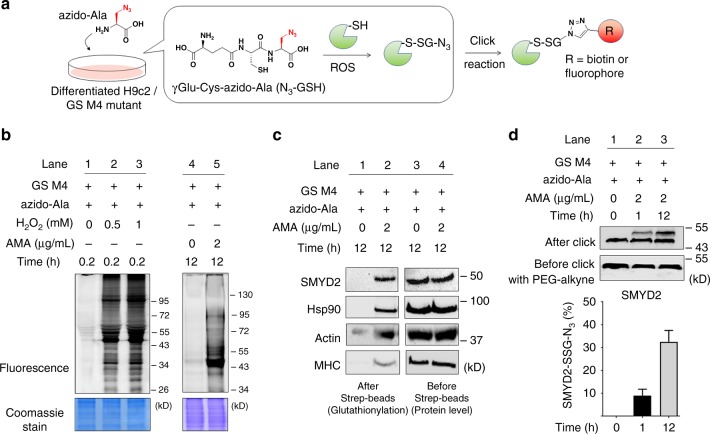
SMYD2 is glutathionylated in response to ROS. **a** A scheme for a clickable glutathione approach: a glutathione synthetase mutant (GS M4), which synthesizes azido-glutathione (γGlu-Cys-azido-Ala, N_3_-GSH), was expressed in differentiated H9c2 cells. After incubation of azido-Ala, cells were subjected to ROS. Glutathionylated proteins in lysates were identified after click reaction. **b** In-gel fluorescence detection of glutathionylated proteins. H9c2 cells expressing GS M4 were incubated with azido-Ala (0.6 mM) for 20 h and treated with H_2_O_2_ or antimycin A (AMA). Collected lysates were then subjected to click reaction with rhodamine-alkyne for fluorescence detection. **c** Identification of individual glutathionylated proteins. Glutathionylated proteins were subjected to click reaction with biotin-alkyne and pull-downs with streptavidin-agarose, and detected by western blotting with individual antibodies, including SMYD2, Hsp90, actin, and myosin-heavy chain (MHC). **d** The level of glutathionylation on SMYD2. Lysates were subjected to click reaction with 2-kD PEG-alkyne. The mass shift of SMYD2 was analyzed by Western blotting. The level of glutathionylation on SMYD2 (SMYD2-SSG-N_3_) was calculated by dividing the amount of glutathionylated SMYD2 (upper band) by a total amount of SMYD2 (upper and bottom bands) in the blot after click reaction. Data represent the mean ± SD. All data are representative of 3 independent experiments

Previously, we found that SMYD2 can be glutathionylated in response to glucose deprivation in HEK293 cells.^22^ To extend our data to cardiomyocytes, we applied our clickable glutathione approach to a H9c2 myoblast cell line (Fig. 1a). GS M4 mutant was expressed into differentiated H9c2 myocytes (Supplementary Fig. 1a). GS M4 expression did not induce significant cell toxicity or alteration of redox systems, such as a thiol-content or levels of redox enzymes in cells (Supplementary Fig. 1b, c). Cells were then incubated with azido-Ala for 20 h and treated with ROS stimuli (Fig. 1b and Supplementary Fig. 2). After the click reaction of lysates with rhodamine-alkyne, in-gel fluorescence analysis showed that global glutathionylation was induced by exposure to an increasing concentration of hydrogen peroxide or antimycin A (AMA) (Fig. 1b), which is known to induce mitochondrial ROS.^2^ The subsequent pull-down analysis after click reaction of lysates with biotin-alkyne detected glutathionylation of SMYD2 (Fig. 1c and Supplementary Fig. 2a–c). In addition, we also detected glutathionylation of other sarcomere-associated proteins, including Hsp90, actin, and myosin heavy chain (MHC) (Fig. 1c and Supplementary Fig. 2a–c). To estimate the level of glutathionylation on SMYD2, lysates were subjected to click reaction with 2 kD-polyethyleneglycol (PEG)-alkyne, which increases the molecular mass of SMYD2. The subsequent western blotting analysis found that over 30% of SMYD2 was glutathionylated upon treatment of AMA for 12 h (Fig. 1d). In the same condition, we did not detect a significant level of irreversible oxidation of SMYD2, such as sulfonic acid formation (Supplementary Fig. 2d). Overall, these data support that SMYD2 is susceptible to glutathionylation in response to ROS.

### SMYD2 is selectively glutathionylated at Cys13

**Fig. 2:**
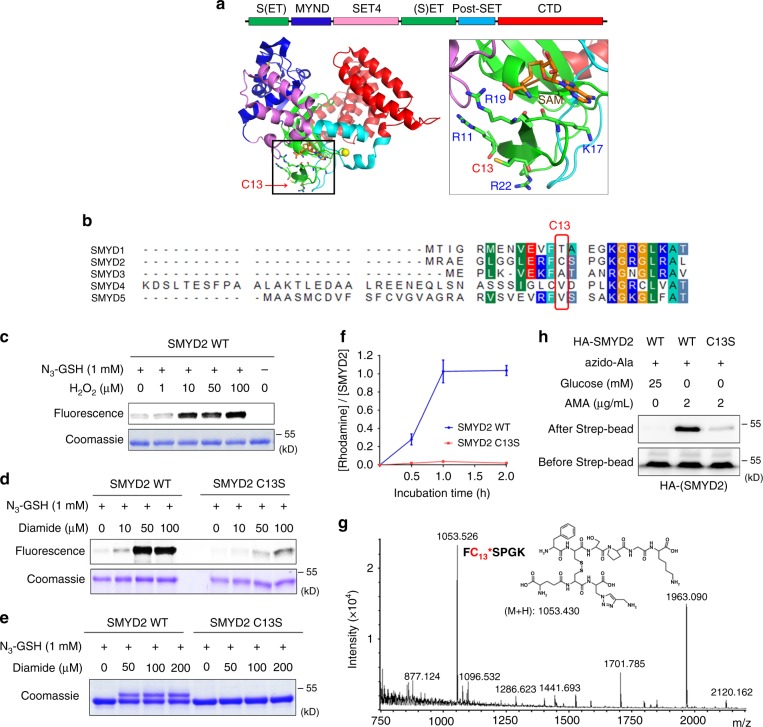
SMYD2 is selectively glutathionylated at Cys13. **a** The structure (PDB: 3RIB) and domains of SMYD2, and an enlarged area around Cys13 in SMYD2. **b** Sequence alignment around Cys13 of SMYD2 with other members of the SMYD family. **c**–**e** In-gel analysis of SMYD2 glutathionylation with azido-glutathione. Purified SMYD2 WT or C13S was mixed with azido-glutathione in vitro and treated with H_2_O_2_ or diamide for 15 min. SMYD2 glutathionylation was detected by fluorescence (**c**, **d**) or a mass shift (**e**) after click reaction with rhodamine-alkyne or 2-kD PEG-alkyne, respectively. **f** Quantifying the molar ratio of rhodamine to SMYD2 concentrations after incubation of SMYD2 with oxidized azido-glutathione (N_3_-GSSG-N_3_) and click reaction with rhodamine-alkyne. Data represent the mean ± SD. **g** Mass spectrometry analysis of glutathionylated SMYD2. SMYD2 glutathionylated by azido-glutathione was conjugated with biotin-alkyne and digested by trypsin. The digested peptides were enriched by streptavidin-agarose and eluted for MALDI-TOF analysis. **h** Detection of SMYD2 Cys13 glutathionylation in HEK293 cells stably expressing GS M4 (HEK293/GS M4). After transfection of SMYD2 WT or C13S, cells were incubated with azido-Ala. After inducing glutathionylation, collected lysates were subjected to click reaction with biotin-alkyne before pull-downs with streptavidin-agarose and western blotting. All data are representative of 3 independent experiments

SMYD2 has 17 cysteine residues, ten of which are bound to three zinc ions in the MYND and Post-SET domains (Fig. 2a).^32^ SMYD2 structural data (PDB: 3RIB) showed that Cys13 is highly exposed at the protein surface and is surrounded by four basic Arg or Lys residues (Fig. 2a, right), which may increase its chemical reactivity. Cys13 is in the SET-domain (Fig. 2a, green) and it is close to a S-adenosylmethionine (SAM) binding site (Fig. 2a). Cys13 in SMYD2 is not conserved among members of the SMYD family (Fig. 2b) but is found in its orthologs (Supplementary Fig. 3). To determine whether Cys13 is important for SMYD2 glutathionylation, we purified wild-type (WT) and C13S mutant proteins of SMYD2 (Supplementary Fig. 4a) and evaluated their glutathionylation with azido-glutathione in vitro. After click reaction with rhodamine-alkyne, in-gel fluorescence analysis showed that SMYD2 WT was strongly glutathionylated upon addition of H_2_O_2_ or diamide (Fig. 2c, d and Supplementary Fig. 5a), whereas SMYD2 C13S showed weak signals (Fig. 2d). In addition, the same result was observed when click reaction was done with 2-kD PEG-alkyne, showing one Cys modification with SMYD2 WT versus no modification with SMYD2 C13S (Fig. 2e and Supplementary Fig. 5b–c). Furthermore, the level of glutathionylation was quantified by measuring the molar ratio of rhodamine to SMYD2 concentrations after click reaction with rhodamine-alkyne (Fig. 2f and Supplementary Fig. 5d). Incubation of SMYD2 with oxidized azido-glutathione (N_3_-GSSG-N_3_) led to an approximately 1:1 molar ratio of rhodamine to SMYD2 (Fig. 2f, blue). In contrast, almost no modification was observed with SMYD2 C13S (Fig. 2f, red), showing the selective glutathionylation at Cys13 of SMYD2.

To directly confirm the modification site, we analyzed glutathionylated SMYD2 by mass spectrometric analysis: SMYD2 glutathionylated by azido-glutathione was conjugated with biotin-alkyne by click reaction and digested by trypsin. The glutathionylated peptides were purified by streptavidin-beads. MALDI analysis of eluted samples found one peak that is in precise agreement with the molecular weight of the peptide glutathionylated at Cys13 (m/z 1053.5, FC^*^SPGK) (Fig. 2g and Supplementary Fig. 6a–b). LC-MS/MS analysis confirmed this assignment and found two additional peptides glutathionylated at Cys74 and Cys321 (Supplementary Fig. 6c). Glutathionylation of SMYD2 at Cys13 was further confirmed in HEK293 cells expressing GS M4 (HEK293/GS M4). In response to AMA with glucose deprivation,^22^ SMYD2 WT was strongly glutathionylated, whereas glutathionylation was significantly decreased with SMYD2 C13S (Fig. 2h). Taken together, our data support that SMYD2 is selectively glutathionylated at Cys13.

### SMYD2 glutathionylation or oxidation decreases cell viability

**Fig. 3:**
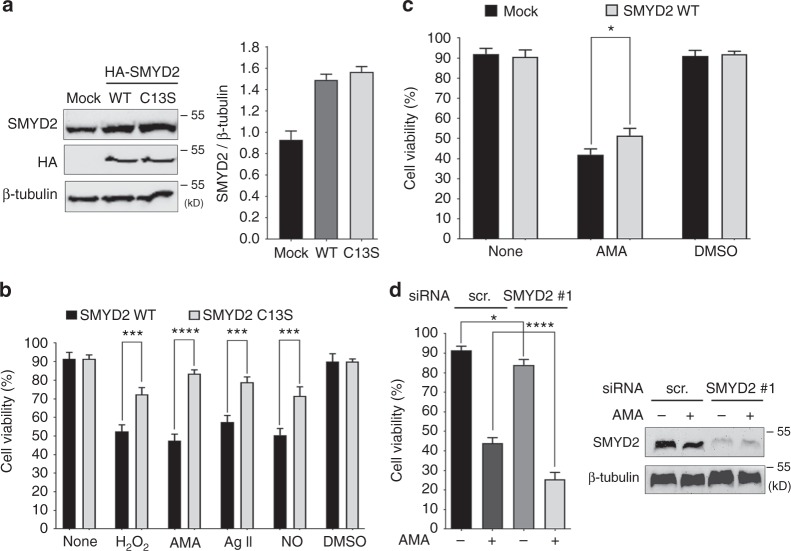
SMYD2 Cys13 glutathionylation or oxidation decreases cell viability. **a** Levels of SMYD2 WT and C13S in differentiated H9c2 cells. **b** Viability of differentiated H9c2 cells expressing SMYD2 WT or C13S after exposure to H_2_O_2_ (25 µM), AMA, (2 µg/mL), angiotensin II (Ag II, 1 µM), or a nitric oxide (NO) donor (NONOate, 100 µM) for 24 h. **c**, **d** Viability of differentiated H9c2 cells with overexpression (**c**) or knockdown (**d**) of SMYD2 after treatment of AMA (2 µg/mL) for 24 h. Data represent the mean ± SD, *n* = 3 independent experiments. Difference is significant by two-way ANOVA followed by Bonferroni’s post-hoc test (**b**, **c**) and one-way ANOVA followed by Tukey’s post-hoc test (**d**), *p < 0.05, **p < 0.01, ***p < 0.001, ****p < 0.0001

Despite a dispensable role of SMYD2 in mouse heart development,^33^ we hypothesized that SMYD2 may have an important role in muscle under stressed conditions. To examine the functional significance of SMYD2 Cys13 glutathionylation, we compared cell viability of H9c2 myoblasts following expression of SMYD2 WT versus C13S, differentiation to a cardiomyocyte phenotype, and subsequent exposure to various ROS stimuli (Fig. 3a, b). After differentiation, SMYD2 levels were similar in the two cohorts of cells expressing SMYD2 WT or C13S (49–50% transfection efficiency, Supplementary Fig. 7a) within 1.7-fold of the endogenous level (Fig. 3a). Sarcomeric α-actinin was also expressed in a similar level (Supplementary Fig. 7b). Differentiated H9c2 cells were then treated with H_2_O_2_ (25 µM), AMA (2 µg/mL), a nitric oxide donor (NONOate, 100 µM), or angiotensin II (1 µM) (Fig. 3b). Under unstressed conditions, viability was comparable in cells expressing SMYD2 WT or C13S (Fig. 3b, bars 1–2 and 11–12). However, in all stressed conditions, viability was significantly decreased in cells expressing SMYD2 WT versus C13S (Fig. 3b, bars 3–10), suggesting that SMYD2 Cys13 glutathionylation or oxidation decreases cell viability.

To corroborate these findings, we examined cell viability after SMYD2 overexpression or knockdown. There is no difference of cell viability in an unstressed condition after SMYD2 overexpression (Fig. 3c, bars 1 vs. 2). However, SMYD2 overexpression rescued viability of cells treated with AMA in a modest but statistically significant level (Fig. 3c, bars 3 vs. 4). After SMYD2 knockdown, there is a modest reduction of cell viability in an unstressed condition (without AMA) (Fig. 3d, bars 1 vs. 3). However, SMYD2 knockdown sensitized cells to treatment of AMA, inducing more significant reduction of cell viability (Fig. 3d, bars 2 vs. 4, and Supplementary Fig. 10a–b). Overall, these results suggest that SMYD2 overexpression provides cellular protection under cellular stress, and a loss of SMYD2 or SMYD2 glutathionylation (or oxidation) in response to ROS decreases viability of H9c2 cells.

### SMYD2 glutathionylation reduces myofibril integrity

**Fig. 4:**
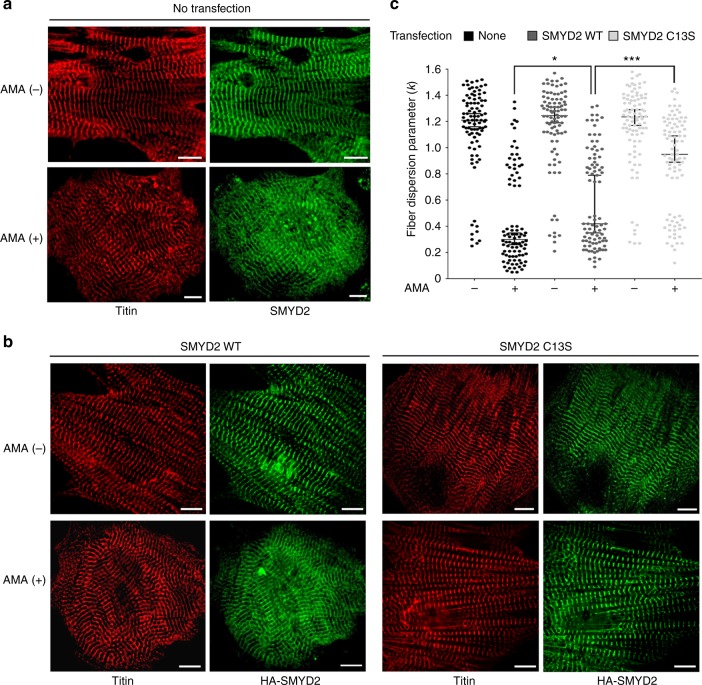
SMYD2 Cys13 glutathionylation or oxidation reduces myofibril integrity. **a**, **b** Monitoring the myofibril alignment in rat neonatal cardiomyocytes upon incubation of AMA (2 µg/mL) for 12 h: no expression (**a**) and ectopic expression of SMYD2 WT or C13S (**b**). Immunostainings were done by using antibodies to SMYD2, HA (green), or titin (α-titin-NT, red). About 30 cells were photographed and examined for myofibril alignment or directionality by FiberFit software.^34^ Images represent the major myofibril structure in individual conditions. Scale bars, 10 µm. **c** Analysis of myofibril alignment in cardiomyocytes. Individual cell images were analyzed by the FiberFit software to determine the fiber dispersion parameter (*k*) values that represent the degree of fiber alignment. High *k* values represent the aligned networks, whereas low *k* values represent the disordered networks. The median values with 95% CI are shown, *n* = 3 independent experiments. Difference is significant by one-way ANOVA, followed by Tukey’s post-hoc test, *p < 0.05, **p < 0.01, ***p < 0.001, ****p < 0.0001

Because SMYD2 is reportedly involved in sarcomere stabilization,^30^ we proposed that the reduced cell viability may be associated with altered sarcomere stability upon SMYD2 glutathionylation. To monitor myofibril structure under stress, we examined rat neonatal cardiomyocytes treated with AMA. Immunostaining of titin, with antibody that binds to the N-terminal region of titin (α-titin-NT), showed a parallel array of striated myofibrils in unstressed cardiomyocytes (Fig. 4a, top left). Directionality analysis by Fiberfit software^34^ showed uniform orientation of myofibrils (Supplementary Fig. 8a) with relatively high fiber dispersion parameter (*k*) values that indicate the high degree of fiber alignment^34^ (Fig. 4c, lane 1, *n* = 30, triplicate). In contrast, upon incubation of AMA, myofibrils were highly misaligned with a complete loss of directionality (Fig. 4a, bottom left). Directionality analysis confirmed the same result, showing a loss of myofibrillar structural integrity (Supplementary Fig. 8a) with low *k* values that indicate the low degree of fiber alignment^34^ (Fig. 4c, lane 2, *n* = 30, triplicate). Immunostaining of SMYD2, which binds to the N2A domain of titin, showed the same pattern while showing high levels of colocalization with titin (Fig. 4a, right).

To examine the importance of SMYD2 Cys13 glutathionylation or oxidation for myofibrillar structure, we repeated experiments upon expression of SMYD2 WT or C13S to rat neonatal cardiomyocytes. Without treatment of AMA, both cells expressing SMYD2 WT or C13S showed parallel striated myofibrils stained by titin or HA antibodies (Fig. 4b, top row) with high *k* values in directionality analysis (Fig. 4c, lanes 3 and 5, *n* = 30, triplicate). However, after incubation of AMA for 12 h, cells with SMYD2 WT showed disoriented and misaligned myofibrils (Fig. 4b, bottom, columns 1–2). Strikingly, cells with SMYD2 C13S retained parallel and regular arrangement of myofibrils in a similar pattern to that seen in the unstressed condition (Fig. 4b, bottom, columns 3–4). Directionality analyses of myofibrils in individual cells showed that treatment of AMA induced misaligned myofibrils in a high number of cells expressing SMYD2 WT versus a low number of cells expressing SMYD2 C13S (Fig. 4c. lanes 4 vs. 6, *n* = 30 cells, triplicate, and Supplementary Fig. 8b–c). In a similar manner, we also examined the structural integrity of differentiated H9c2 cells by fluorescence imaging of EGFP-actin, mCherry-MHC, and α-actinin immunostaining. All analyses showed the same pattern that upon treatment of AMA, cells expressing SMYD2 WT showed a loss of structural integrity, whereas cells with SMYD2 C13S maintained the similar structural integrity to ones in the unstressed condition (Supplementary Fig. 7c–d). Overall, these data support the concept that SMYD2 Cys13 glutathionylation or oxidation contributes to misalignment or destabilization of myofibrils.

### SMYD2 glutathionylation leads to sarcomeric proteins degradation

**Fig. 5:**
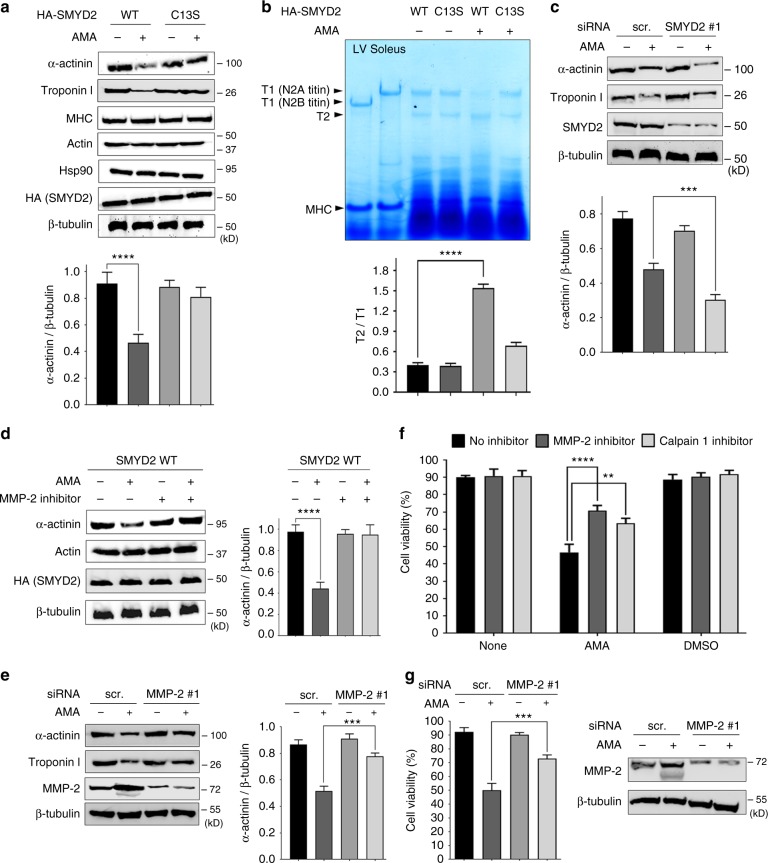
SMYD2 Cys13 glutathionylation or oxidation leads to degradation of sarcomeric proteins. **a** Sarcomeric protein levels in response to AMA in differentiated H9c2 cells that express SMYD2 WT or C13S. **b** Levels of titin in response to AMA in HL-1 cells expressing SMYD2 WT or C13S. Extracts of left ventricle (LV) and soleus muscle isolated from 6.5-months old rat were used as standards (lane 1 and lane 2) to show the position of N2B-titin or N2A-titin isoforms, respectively. **c** Sarcomeric protein levels in response to AMA after SMYD2 knockdown. **d**, **e** Sarcomeric protein levels in response to AMA after incubation of ARP-100 (MMP-2 inhibitor) (**d**) or MMP-2 knockdown (**e**). **f**, **g** The cell viability in response to AMA after incubation of ARP-100 (1 µM), calpastatin (calpain 1 inhibitor, 5 µM) (**f**) or MMP-2 knockdown (**g**). In all conditions, differentiated H9c2 (**a**, **c**–**g**) or HL-1 cells (**b**) were treated with AMA (2 µg/mL) for 12 h. Lysates were analyzed by Western blotting (**a**, **c**–**e**, **g**) or Coomassie staining (**b**). Cell viability was analyzed by Trypan blue assay. Data represent the mean ± SD, *n* = 3 independent experiments. Difference is significant by two-way ANOVA followed by Bonferroni’s post-hoc test (**f**) and one-way ANOVA followed by Tukey’s post-hoc test (**a**–**e**, **g**), *p < 0.05, **p < 0.01, ***p < 0.001, ****p < 0.0001

The loss of stability or integrity of myofibrils is likely due to degradation of sarcomeric proteins. Therefore, we compared protein levels in H9c2 cells expressing SMYD2 WT versus C13S (Fig. 5a). Before treatment with AMA, the protein level of α-actinin was similar in differentiated H9c2 cells expressing SMYD2 WT or C13S (Supplementary Fig. 7b). Exposure to AMA resulted in decreased levels of α-actinin and troponin I in cells with SMYD2 WT (Fig. 5a, lane 1 vs. 2), but had no effect in cells with SMYD2 C13S (Fig. 5a, lane 3 vs. 4). Under the same condition, treatment with AMA had no effect on the expression levels of actin, MHC, SMYD2, and Hsp90 in cells with SMYD2 WT or C13S (Fig. 5a). In addition to H9c2 cells, we also examined HL-1 mouse cardiac muscle cells upon ectopic expression of SMYD2 WT or C13S. Similarly, reduced levels of α-actinin and troponin I were observed in cells expressing SMYD2 WT, whereas no changes were observed in cells with SMYD2 C13S (Supplementary Fig. 9a). Next, we examined the level of titin. Without treatment of AMA, the level of titin (T1) was high in HL-1 cells expressing SMYD2 WT or C13S (Fig. 5b, lane 3 and 4). However, upon treatment of AMA, the level of T2, a degraded product of titin^35^, was increased in cells expressing SMYD2 WT (Fig. 5b, lane 5), whereas it remained low in cells with SMYD2 C13S (Fig. 5b, lane 6). To further support degradation of titin, we examined titin by dot-blot analysis after the immunoprecipitation (IP): the blot was probed with α-titin-NT antibody, which recognizes the N-terminal region of titin, after pull-down of titin with α-titin-CT antibody, which binds to the C-terminal region of titin (Supplementary Fig. 9b–c). Upon treatment of AMA, the level of titin detected by α-titin-NT was decreased in cells expressing SMYD2 WT, whereas it was unchanged in cells expressing SMYD2 C13S (Supplementary Fig. 9b–c), suggesting degradation of titin in cells expressing SMYD2 WT versus C13S. In addition to sarcomeric proteins, we also found that cardiac ankyrin repeat protein (CARP), transcription cofactor that is involved in muscle remodeling,^36^ was highly elevated in cells with SMYD2 WT versus C13S after treatment of AMA (Supplementary Fig. 9d).

To corroborate these findings, we examined the level of sarcomeric proteins upon SMYD2 knockdown. Without treatment of AMA, levels of α-actinin or troponin I remain similar upon SMYD2 knockdown (Fig. 5c, lane 1 vs. 3). However, SMYD2 knockdown induced more significant reduction of α-actinin and troponin I levels upon incubation of AMA (Fig. 5c, lane 2 vs. 4, and Supplementary Fig. 10c), which correlates with the data that SMYD2 knockdown induces more significant reduction of cell viability after incubation of AMA (Fig. 3d, bars 2 vs. 4, and Supplementary Fig. 10a–b). These data support that SMYD2 knockdown or glutathionylation (or oxidation) at Cys13 contributes to reduced levels of several sarcomeric proteins, such as α-actinin, troponin I, and titin.

Previously, calpain 1/3 and MMP-2 have been found to play a role in degradation of sarcomeric proteins, including α-actinin and titin.^14,15^ Therefore, we evaluated whether calpain and MMP-2 contribute to sarcomeric protein degradation in our model. The decreased levels of α-actinin and troponin I in the presence of AMA were restored upon incubation with an MMP-2 inhibitor (ARP-100) (Fig. 5d, lane 2 vs. 4) or MMP-2 knockdown (Fig. 5e, lane 2 vs. 4, and Supplementary Fig. 10d), while there was no effect on the expression levels of other proteins, including actin and SMYD2 (Fig. 5d). Notably, levels of the native MMP-2 and its smaller-size form (either proteolytic or inducible form^37^) were significantly elevated upon incubation of AMA (Fig. 5e, g), which is consistent with the fact that MMP-2 is activated under oxidative stress.^12^ However, overexpression of a truncated active form of MMP-2, without treatment of AMA, did not induce significant degradation of α-actinin (Supplementary Fig. 10e), suggesting that the stressor as well as active MMP-2 may be necessary for significant degradation of sarcomeric proteins.

We further evaluated H9c2 cell viability upon exposure to inhibitors of MMP-2 (ARP-100) and calpain 1 (acetyl-calpastatin) (Fig. 5f) or MMP-2 knockdown (Fig. 5g). As expected, viability of H9c2 cells was significantly decreased after treatment of AMA (Fig. 5f, g). This effect was mitigated by treatment with either the MMP-2 or calpain 1 inhibitor (Fig. 5f, bars 4 vs. 5 and 6) or MMP-2 knockdown (Fig. 5g, bars 2 vs. 4), suggesting that calpain 1 and MMP-2 contribute to the stressor-induced reduction of cell viability. Overall, these data reveal that SMYD2 C13S is protective against sarcomeric proteins degradation and cell death in oxidatively stressed conditions, and that SMYD2 Cys13 glutathionylation or oxidation contributes to degradation of sarcomeric proteins mediated by MMP-2 and calpain-1.

### SMYD2 glutathionylation dissociates SMYD2 from N2A

In myocytes, SMYD2 is involved in mono-methylation of Hsp90, which increases Hsp90 chaperone activity.^31^ SMYD2 then forms a complex with mono-methylated Hsp90.^31^ This complex binds to N2A, a domain of titin, which has been implicated to be important for sarcomere stabilization.^30^ Therefore, we investigated whether glutathionylation of SMYD2 changes its enzymatic activity or interaction with Hsp90 or the N2A domain. To interrogate the effect of glutathionylation on SMYD2 in vitro, purified SMYD2 WT was glutathionylated by incubating with oxidized glutathione (GSSG) (Supplementary Fig. 11a). Note that similar incubation of SMYD2 with oxidized azido-glutathione (N_3_-GSSG-N_3_) induced selective glutathionylation of SMYD2 at Cys13 (Fig. 2f). SMYD2 WT (SMYD2-SH) and glutathionylated SMYD2 (SMYD2-SSG) were further purified (Supplementary Fig. 11b–d). SMYD2-SH and SMYD2-SSG show the same partial digestion pattern by trypsin (Supplementary Fig. 11e), suggesting their similarly folded structures. SMYD2 enzymatic activity was then examined by LC-MS methylation analysis^38^ (Supplementary Fig. 12). In this assay, when using Hsp90 as a substrate, S-adenosylhomocysteine (SAH) production was decreased by approximately 50% with SMYD2-SSG versus SMYD2-SH (the rate of SAH production: 13.2 ± 1.6 nM min^−1^ with SMYD2-SH vs. 7.4 ± 1.0 nM min^−1^ with SMYD2-SSG) (Supplementary Fig. 12a). In comparison, when p53 peptide was used as a substrate, SAH production was less significantly decreased with SMYD2-SSG versus SMYD2-SH (Supplementary Fig. 12b). Neither N2A nor MMP-2 was a substrate of SMYD2 (Supplementary Fig. 12c and 14). Despite the modest reduction of SMYD2 enzymatic activity upon glutathionylation in vitro, the level of Hsp90 methylation was unchanged in cells upon treatment of AMA (Supplementary Fig. 13a). Importantly, SMYD2 Y240F (catalytically inactive mutant)^39^ could also lead to degradation of α-actinin in the same manner to SMYD2 WT (Supplementary Fig. 13c). Conversely, SMYD2 C13S/Y240F mutant prevented degradation of α-actinin similarly to SMYD2 C13S (Supplementary Fig. 13c). These data suggest that SMYD2 enzyme activity is unlikely responsible for sarcomeric protein degradation.

**Fig. 6:**
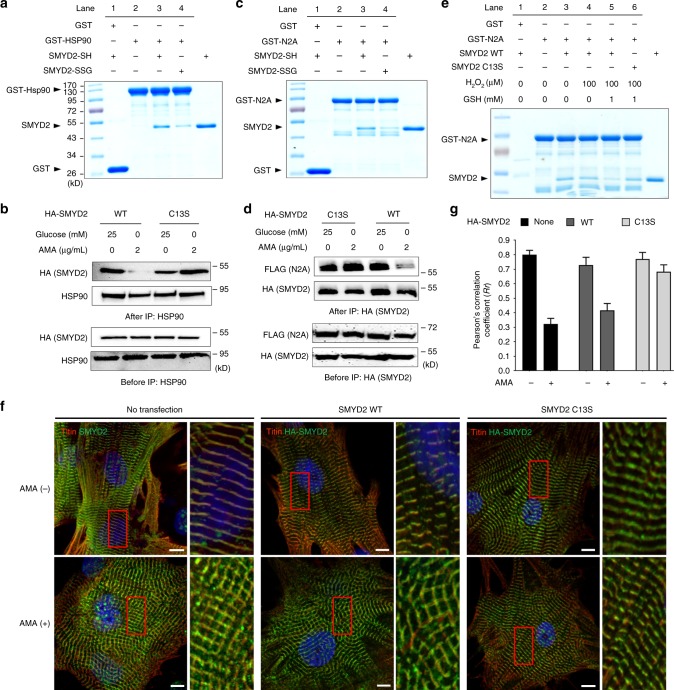
SMYD2 Cys13 glutathionylation induces dissociation of SMYD2 from N2A and Hsp90. **a**, **b** SMYD2 glutathionylation disrupts its interaction with Hsp90. Purified SMYD2-SH and SMYD2-SSG were incubated with GST-Hsp90 bound to glutathione beads, and eluted sample was analyzed (**a**). Hsp90 was co-immunoprecipitated with SMYD2 WT or C13S from HEK293 cells in response to AMA with glucose deprivation (**b**). **c**, **d** SMYD2 glutathionylation disrupts its interaction with N2A. Purified SMYD2-SH and SMYD2-SSG were incubated with GST-N2A bound to glutathione beads, and eluted sample was analyzed (**c**). FLAG-N2A was co-immunoprecipitated with SMYD2 WT or C13S in HEK293 cells in response to AMA with glucose deprivation (**d**). **e** SMYD2 subjected to glutathionylation decreases its binding with N2A. SMYD2 WT or C13S was pre-incubated with H_2_O_2_ in the absence or presence of glutathione for 15 min, then mixed with GST-N2A bound to glutathione beads for 1 h. Eluted samples were analyzed. **f**, **g** Colocalization of titin and SMYD2 decreases upon incubation of AMA in rat neonatal cardiomyocytes expressing SMYD2 WT versus C13S. Immunostainings of cardiomyocytes with antibodies to titin (α-titin-NT, red), HA, or SMYD2 (green) are shown with enlarged areas for details (the red boxes) (**f**). Pearson’s correlation coefficients were calculated to determine colocalization of titin and SMYD2 (**g**). About 30 cells were analyzed in individual conditions. Images represent the major colocalization pattern in individual experiments. Scale bars, 10 µm. Data represent the mean ± SD, *n* = 3 independent experiments

Next, we analyzed whether SMYD2–Hsp90–N2A interactions can be disrupted upon SMYD2 glutathionylation. A binding assay showed that GST-Hsp90 binds to SMYD2-SH more strongly than SMYD2-SSG (Fig. 6a, lane 3 vs. 4), showing that SMYD2 glutathionylation decreases the SMYD2–Hsp90 interaction. We also examined the SMYD2–Hsp90 interaction by co-immunoprecipitation (co-IP) in HEK293 cells expressing SMYD2 WT or C13S (Fig. 6b): SMYD2 WT binding with Hsp90 was significantly decreased in stressed conditions (AMA and glucose deprivation) (Fig. 6b, lane 1 vs. 2). In contrast, SMYD2 C13S interaction with Hsp90 did not change in identical stressed conditions (Fig. 6b, lane 3 vs. 4). Similarly, the N2A domain was examined for binding with SMYD2 (Fig. 6c, d). SMYD2 binding to N2A was decreased with SMYD2-SSG versus SMYD2-SH (Fig. 6c, lane 3 vs. 4). Co-IP experiments showed that SMYD2 WT lost its interaction with N2A in the presence of stressors (Fig. 6d, lane 3 vs. 4) while SMYD2 C13S retained its interaction (Fig. 6d, lane 1 vs. 2).

To further demonstrate that SMYD2 is dissociated from the N2A domain or titin upon SMYD2 glutathionylation or oxidation, we examined colocalization of SMYD2 and titin in the sarcomere of rat neonatal cardiomyocytes expressing SMYD2 WT or C13S. Consistently, immunostaining showed high levels of SMYD2 co-localized with titin in the absence of AMA (Fig. 6f, top left). However, colocalization of SMYD2 with titin was decreased upon incubation of AMA (Fig. 6f, bottom left, and Fig. 6g, *n* = 30, Pearson Coefficient 0.80 ± 0.03 and 0.32 ± 0.04 without and with AMA, respectively). Notably, colocalization of SMYD2 with titin was also decreased in cells expressing SMYD2 WT after incubation of AMA (Fig. 6f, middle, and Fig. 6g, *n* = 30, Pearson Coefficient 0.75 ± 0.05 and 0.43 ± 0.04 without and with AMA, respectively), whereas colocalization remains high in cells expressing SMYD2 C13S (Fig. 6f, right, and Fig. 6g, *n* = 30, Pearson Coefficient 0.77 ± 0.05 and 0.67 ± 0.06 without and with AMA, respectively). Taken together, these data provide evidence that SMYD2 Cys13 glutathionylation (or oxidation) disrupts the SMYD2 interactions with Hsp90 and N2A of titin and induces dissociation of SMYD2 from titin and the sarcomere.

In addition to glutathionylation, cysteine oxidation in proteins involves formation of sulfenic acid, sulfinic acid, and sulfonic acid,^40^ which are relatively smaller size modifications in comparison to glutathionylation (Supplementary Fig. 15a). Although we did not detect sulfonic acid formation in SMYD2 (Fig. 2d), we examined potential effects of these oxidations on the interaction of SMYD2 with N2A. In vitro binding analysis between GST-N2A and SMYD2 showed that SMYD2 pre-incubated with a low amount of H_2_O_2_ (0–100 µM) retained its binding with GST-N2A (Fig. 6e, lane 3 vs. 4, and Supplementary Fig. 16a). However, in identical conditions, SMYD2 WT subjected to glutathionylation by pre-incubation with H_2_O_2_ and glutathione lost its interaction with GST-N2A (Fig. 6e, lane 3 vs. 5). In contrast, SMYD2 C13S subjected to glutathionylation in the same condition retained its binding with N2A (Fig. 6e, lane 3 vs. 6), suggesting that SMYD2 C13 glutathionylation is likely responsible for dissociation of the SMYD2–N2A interaction. However, SMYD2 decreased its binding with GST-N2A when pre-incubated with a relatively high amount of H_2_O_2_ (100–500 µM) (Supplementary Figure 16b), suggesting that other types of oxidations in addition to glutathionylation in SMYD2 may also lead to its dissociation from N2A.

To further specify and mimic a small size oxidative modification in SMYD2 at Cys13, we expressed SMYD2 C13D (Supplementary Fig. 15b) in which Asp serves as a close mimic of sulfinic acid at Cys13 with respect to both size and charge (Supplementary Fig. 15a).^41,42^ In vitro binding assays showed that similar levels of SMYD2 WT and C13D were bound to Hsp90 and N2A (Supplementary Fig. 15c–d). Co-IP experiments confirmed these results, showing that SMYD2 WT and C13D maintain the similar level of binding with both Hsp90 and N2A (Supplementary Fig. 15e), suggesting that a small size of modifications at Cys13 of SMYD2 may not disrupt the SMYD2 interaction with N2A or Hsp90.

### SMYD2-N2A dissociation contributes to sarcomeric protein degradation

**Fig. 7:**
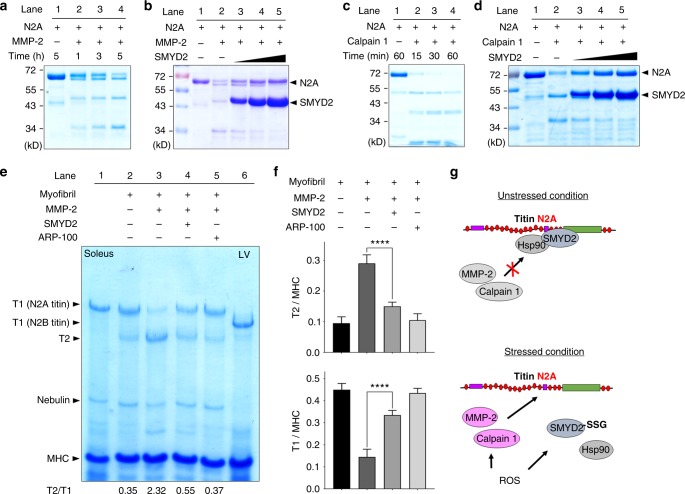
SMYD2-N2A dissociation contributes to degradation of sarcomeric proteins. **a**, **b** N2A is degraded by MMP-2, and SMYD2 protects N2A from degradation. Purified N2A was incubated with active MMP-2 in a time-dependent manner (**a**) or with an increasing amount of SMYD2 (**b**). **c**, **d** N2A is degraded by calpain 1, and SMYD2 protects N2A from degradation. Purified N2A was incubated with calpain 1 in a time-dependent manner (**c**) or with an increasing amount of SMYD2 (**d**). Data are representative of 4 independent experiments. **e**, **f** Titin in isolated myofibrils is degraded by MMP-2, and SMYD2 protects titin from degradation. Myofibrils isolated from mouse gastrocnemius muscle were incubated with active MMP-2 in the absence and presence of SMYD2 (**e**). Extracts of soleus muscle and left ventricle (LV) isolated from 6.5-months old rat were used as standards (lane 1 and lane 6). Levels of titin degradation by measuring the ratio of T1 or T2 to MHC (**f**). In all conditions, proteins were analyzed by Coomassie stains. Data represent the mean ± SD, *n* = 3 independent experiments. Difference is significant by one-way ANOVA followed by Tukey’s post-hoc test, *p < 0.05, **p < 0.01, ***p < 0.001, ****p < 0.0001. **g** A proposed mechanism of sarcomere destabilization upon SMYD2 glutathionylation or oxidation. Under unstressed conditions, SMYD2-Hsp90 binds with and protects N2A of titin (top). Under stressed conditions, ROS lead to activation of MMP-2 and calpain 1 while inducing glutathionylation (or other oxidations) of SMYD2, which is then dissociated from N2A or titin, allowing for sarcomeric protein degradation by MMP-2 and calpain 1. It remains to be analyzed how SMYD2 glutathionylation or oxidation contributes to degradation of α-actinin and troponin I (bottom)

Next, we attempted to examine whether the dissociation between SMYD2 and N2A is responsible for sarcomere degradation. We hypothesized that the N2A domain or its proximal region in titin may contain cleavage sites of MMP-2 or calpain-1, which could be protected when SMYD2 binds to N2A, whereas the SMYD2-N2A dissociation may expose N2A for degradation. Notably, several domains of titin in the I-band of sarcomere, which can be unfolded during contraction and relaxation, were suggested to contain MMP-2 cleavage sites.^15^ However, it is unknown whether N2A contains the cleavage site. The N2A domain is composed of four immunoglobulin domains (Ig80-83) and a unique sequence region, called N2A-Us (also called UN2A), between Ig80 and Ig81 (Supplementary Fig. 17a).^43^ We analyzed whether N2A contains the MMP-2 cleavage site by using the MMP cleavage site prediction tool (CleavPredict).^44^ The tool predicted the presence of several MMP-2 cleavage sequences in N2A, including high probability of cleavage sites around N2A-Us (Supplementary Fig. 17b-c). Indeed, purified N2A was cleaved upon incubation with active MMP-2 (Fig. 7a and Supplementary Fig. 17d). The sizes of the cleaved products (approximately 50 and 35 kD) appear to correlate with the potential cleavage in domains around N2A-Us (Supplementary Fig. 17b–c). Notably, addition of SMYD2 C13S, which binds to the N2A domain, decreased degradation of N2A by MMP-2 in a dose-dependent manner (Fig. 7b). Similarly, N2A was predicted to have the calpain 1 cleavage site (Supplementary Fig. 18a–c).^45^ N2A was also susceptible to degradation by calpain 1 (Fig. 7c and Supplementary Fig. 18d). Incubation with SMYD2 C13S protected N2A from calpain 1-mediated degradation (Fig. 7d). Interestingly, both MMP-2 and calpain 1 resulted in cleaved products of N2A that are of similar sizes (Supplementary Fig. 18e), suggesting that N2A has a local motif vulnerable to degradation by both proteases.

With purified N2A and MMP-2 in vitro, we further analyzed whether oxidized or glutathionylated SMYD2 can protect N2A from the MMP-2 mediated degradation. SMYD2 WT pre-incubated with H_2_O_2_ (100 µM) could still prevent degradation of N2A as does SMYD2 WT untreated with H_2_O_2_ (Supplementary Fig. 16c, lane 3 vs. 5). However, SMYD2 WT pre-incubated with H_2_O_2_ (100 µM) and glutathione, which induces glutathionylation of SMYD2, failed to protect N2A, leading to significant degradation of N2A (Supplementary Fig. 16c, lane 3 vs 7). In contrast, SMYD2 C13S subjected to glutathionylation in the same condition retained the protective effect (Supplementary Fig. 16c, lane 3 vs. 8), supporting that SMYD2 C13 glutathionylation contributes to degradation of N2A.

To further support our hypothesis, we performed similar experiments in which N2A was replaced by myofibrils isolated from mouse gastrocnemius muscle that has an N2A-titin isoform.^46^ Incubation of fresh myofibrils with active MMP-2 induced degradation of titin, decreasing the level of N2A-titin (T1) while increasing the level of T2 (Fig. 7e, lane 2 vs. 3, and Fig. 7f). Notably, incubation of SMYD2 protected titin from MMP-2 mediated degradation (Fig. 7e, lane 3 vs. 4, and Fig. 7f). Titin degradation was also inhibited upon an addition of ARP-100 (Fig. 7e, lane 3 vs. 5, and Fig. 7f). In these experiments, there was no apparent degradation of MHC (Fig. 7e). Overall, these data support our model that the SMYD2 binding interaction with N2A, titin, or sarcomeric proteins confers the protection against proteases, whereas SMYD2 dissociation upon glutathionylation or oxidation contributes to degradation of sarcomeric proteins (Fig. 7g).

## Discussion

The detrimental role of ROS in cardiac muscle has been extensively analyzed in ischemia-reperfusion injuries that are well-known to cause a burst of mitochondrial ROS and contribute to muscle damage.^47–50^ Many of these ROS effects partially result from oxidative modifications of sarcomeric or myofibrillar proteins.^51–55^ For example, ROS elevated during ischemic reperfusion cause glutathionylation and carbonylation of actin,^52^ glutathionylation of troponin subunits,^56^ and disulfide formation in tropomyosin,^51^ nitration of myosin,^53^ many of which result in the reduced contractile force of myofilaments. Titin is also oxidized in multiple regions. For example, the N2B domain of titin forms disulfide, which increases muscle stiffness.^43^ The cryptic cysteine residues in Ig-domains of titin at the I-band are also glutathionylated, which reduces passive stiffness.^57^

In addition to reduction of myofilament contraction, numerous data support that cellular stress during ischemic-reperfusion or nutrient starvation causes proteolysis of sarcomeric and myofibrillar proteins.^12,13^ There is emerging evidence that the highly ordered structure of sarcomere is maintained in a dynamic process that involves an intricate balance between assembly and degradation of sarcomeric proteins by the action of many chaperones and proteases.^14^ However, the molecular link between sarcomeric protein oxidative modification and the action of the protease system is still incompletely understood. In this report, we showed that glutathionylation and potentially other types of oxidations of sarcomere-associated SMYD2 serves as a mechanism of ROS that contributes to degradation of sarcomeric proteins.

In this report, we used our clickable glutathione approach to detect glutathionylation of multiple proteins, including SMYD2, under stressed conditions. A key idea of our approach is routing glutathione biosynthesis to clickable glutathione by using a mutant of a glutathione biosynthetic enzyme.^58^ An azide-tag on glutathione can be replaced by other bioorthogonal functional groups, including terminal-alkene.^21^ A modified clickable glutathione is an efficient substrate of several redox enzymes, and is tolerated in cells without significant disturbance of the redox system (Supplementary Fig. 1),^21,22^ all of which support that our approach is suitable for investigating glutathionylation in response to cellular stress.

We confirmed glutathionylation of SMYD2 in various stressed conditions (Fig. 1 and Supplementary Fig. 2) and found selective glutathionylation at Cys13 (Fig. 2). While there are 17 Cys residues in SMYD2, many of them are bound to zinc atoms or buried inside SMYD2, thus may not be accessible for glutathionylation. In our experiments, we did not detect sulfonic acid formation in SMYD2 (Supplementary Fig. 2d). However, other oxoforms, such as sulfenic acid, may form. It is also possible to form Cys modifications with other electrophiles, such as 4-hydroxynonenal^59^ and fumarate,^60^ which may induce a similar cellular phenotype to glutathionylation.

A key observation in our report is that myofibril integrity is lost in cells expressing SMYD2 WT in response to ROS, whereas SMYD2 C13S protects myofibrils from degradation (Fig. 4), showing an important role of SMYD2 glutathionylation or oxidation at Cys13 in myofibril integrity or sarcomere stability. While our data may suggest a pathologic consequence of SMYD2 oxidation or glutathionylation in muscle, sarcomere degradation or disassembly is not only found in pathologic conditions, such as cardiomyopathy.^61,62^ Sarcomere degradation is also observed in physiologic processes during muscle growth or remodeling that requires partial degradation of sarcomeres or myofibrils in order to form a higher mass of muscle.^63^ Indeed, the beneficial role of sarcomere proteolysis is well-recognized in skeletal muscle growth and stress adaptation.^63^ It remains to be seen whether SMYD2 glutathionylation is implicated in regulation of sarcomere degradation in physiologic processes.

Another important finding is that protein interaction between SMYD2 and the N2A domain of titin contributes to modulating myofibril or sarcomere degradation. Indeed, protein-protein interactions at titin’s extensible domains, including N2B, PEVK, and N2A at the I-band, plays a central role in stress-signaling.^16^ For example, the N2B domain has four Ig-domains and one extensible unique sequence region (N2B-Us). A small chaperone, αB-crystallin, binds to N2B-Us for stabilization or protection of sarcomere from stress.^64^ N2B-Us also interacts with signaling complexes, including four-and-a-half-LIM-domain protein (FHL2) that can translocate to the nucleus to participate in gene expression.^16^ Similarly, N2A has four Ig-domains and one extensible unique sequence (N2A-Us). Hsp90-SMYD2 chaperone complex binds to N2A.^30^ Our data showed that the SMYD2-N2A interaction protects N2A from degradation by MMP-2 and calpain 1 (Fig. 7a–d), and SMYD2 also protect titin in myofibrils from MMP-2 mediated cleavage (Fig. 7e, f). Therefore, our data support the concept that N2A is an important domain of titin that the SMYD2–Hsp90 chaperone complex interacts with for stabilization or protection of sarcomeres.

## Methods

### Cell culture

H9c2 cells (ATCC, CRL-1446) were cultured in DMEM supplemented with 10% FBS, penicillin (100 units/mL) and streptomycin (100 μg/mL). HL-1 cells (Sigma, SCC065) were cultured in Claycomb medium (Sigma, 51800 C) supplemented with 10% FBS, penicillin (100 units/mL), streptomycin (100 μg/mL), norepinephrine (0.1 mM), and L-glutamine (2 mM) in fibronectin–gelatin-coated flasks. HL-1 cells were tested negative when examined by MycoAlert mycoplasma detection kit (Lonza). Neonatal rat ventricular cardiomyocytes (P1-2) (Lonza, R-CM-561) were cultured on nitrocellulose-coated plates with the provided medium (Lonza, CC-4515). Cells were cultured at 37 °C in 5% CO_2_ humidified atmosphere.

### Differentiation and induction of glutathionylation

H9c2 cells were differentiated by incubating with differentiation medium (DMEM containing 1% FBS and 1 μM trans-retinoic acid). After 5 days, cells were incubated with DMEM containing 2% FBS, and infected with adenovirus-expressing GS M4 (Ad/GS M4) (custom made from Vector Biolabs) for 6 h. Cells were then incubated with DMEM containing 10% FBS for 18 h, and further incubated for 20 h after addition of L-azido-Ala (0.6 mM). After serum starvation for 4 h, cells were treated with H_2_O_2_ or AMA and lysed with a lysis buffer [100 mM Tris-HCl, 150 mM NaCl, pH 7.4, 0.1% Tween 20, a protease inhibitor cocktail tablet, 100 µM PMSF and 50 mM N-ethylmaleimide (NEM)]. Lysates were collected and analyzed for protein concentration by Bradford assay. Glutathionylation in HEK293 cells stably expressing GS M4^22^ were analyzed in a similar manner after transfection of HA-SMYD2 WT or C13S using Polyethyleneimine (PEI)-MAX (Polysciences, Inc).

### Detection and pull-down of glutathionylated proteins

Proteins in lysates were precipitated by cold acetone, re-dissolved in PBS, and subjected to click reaction with biotin-alkyne, rhodamine-alkyne, or Cy5-alkyne (0.2 mM), CuBr (1 mM) and Tris(3-hydroxypropyltriazolylmethyl)amine (THPTA) (0.4 mM) for 1 h. After separation by SDS-PAGE, proteins in gels were analyzed by Coomassie stains or FluorChem Q imaging system (Biorad). For pull-down of glutathionylated proteins, the click reaction mixture with biotin-alkyne was subjected to precipitation by cold acetone. Proteins were re-dissolved in TBS (100 mM Tris-HCl and 150 mM NaCl) containing 1% SDS with sonication and incubated with pre-washed streptavidin-agarose beads for 3 h at room temperature. After washing with TBST (5 mL × 3), proteins on beads were eluted with an SDS-loading buffer, separated by SDS-PAGE, and analyzed by Western blotting. For mass-shift analysis of SMYD2, lysates were treated in the similar manner, except that click reaction was performed twice with 2-kD PEG-alkyne for 2 h at 37 °C.

### In vitro glutathionylation of SMYD2

To purified SMYD2 WT or C13S in 50 mM PBS containing azido-glutathione (1 mM) was added different concentrations of H_2_O_2_ or diamide (0–100 μM). After 5 min at room temperature, glutathionylation was quenched by addition of iodoacetamide (20 mM) for 15 min at 37 °C in the presence of 1% SDS. Glutathionylation was analyzed, as described above, after click reaction with rhodamine-alkyne (0.2 mM) or 2-kD PEG-alkyne (0.4 mM), THPTA (0.8 mM), CuBr (1 mM) for 1 h at 37 °C. To measure the level of rhodamine-conjugation in SMYD2, SMYD2 was precipitated after click reaction and resuspended in PBS. SMYD2 concentration was determined by comparison to BSA standard while the rhodamine concentration was determined by absorbance at 550 nm (a molar extinction coefficient, 65,000 M^−1^cm^−1^).

### Mass identification of SMYD2 glutathionylation

After inducing glutathionylation of SMYD2 in vitro, SMYD2 was subjected to click reaction with biotin-DDE-alkyne (click chemistry tools) (400 µM), THPTA (2 mM), and Cu(I)Br (2 mM) at room temperature. Proteins were precipitated by cold acetone and washed with cold methanol. Proteins were dissolved in a denaturation buffer (2 M urea, 1 mM CaCl_2_ in PBS) and digested by trypsin overnight at 37 °C. The digest solution was added to streptavidin-agarose beads (Invitrogen) (50 µL) in PBS and incubated for 2 h. Beads were then washed with PBS containing 0.2% SDS (5 mL), followed by PBS only (3 × 5 mL). Bound peptides were then eluted by 2% hydrazine in PBS (3 × 30 µL, pH 7.4). Eluted fractions were combined and acidified with 5% formic acid. The sample was desalted by C18 silica zip-tip (Millipore) and analyzed by Bruker MALDI-TOF/TOF ultrafleXtreme instrument with α-cyano-4-hydroxycinnamic acid as the matrix. FlexAnalysis software was used to assign fragments and to measure distance between *b* and *y* ion series with addition of a modified mass (+415 Da) on cysteine residues. For ESI MS/MS analysis, eluted peptides were separated by UHPLC reverse phase chromatography with an EASY-nLC 1000 liquid chromatography system (Thermo) and introduced into an Orbitrap FUSION mass spectrometer (Thermo). MS1 scans were between 350–1600 m/z and at 240,000 orbitrap resolution. Abundant peptides with +2 or +3 charges were fragmented by CID at 35% collision energy, and peptides with charges between +3 and +7 were fragmented by ETD using calibrated charge-dependent ETD parameters. All MS2 fragmentations were scanned at 30,000 orbitrap resolution. Data were analyzed with the Sequest search algorithm using Proteome Discoverer (ver 2.1) and a human protein database (20,145 entries, downloaded from Uniprot on 2017-07-14). Parent and fragment tolerances were 10 ppm and 0.02 Da, respectively. Variable modifications included oxidation of M (+16) or azido-glutathione modification of C (+415). Tryptic cleavage and up to 1 missed cleavage were used in the search, and data was concurrently searched against a decoy database. Search results were imported into Scaffold (ver 4.8) and re-analyzed with X!Tandem. Protein probabilities were assigned by the Protein Prophet algorithm.^65^ Proteins that contained similar peptides and could not be differentiated based on MS/MS analysis alone were grouped to satisfy the principles of parsimony. Protein thresholds were put at 98% with a minimum number of peptides set to 1.

### Knockdown of SMYD2 and MMP-2

During differentiation, H9c2 cells were transfected twice with SMYD2 siRNA (100 nM) (siRNA #1, Santa Cruz Biotechnology, sc-76530; siRNA #2 Dharmacon, Cat# M-083374-01-0005) or MMP-2 siRNA (100 nM) (siRNA #1, Santa Cruz Biotechnology, sc-37264; siRNA #2, Dharmacon, Cat# M-093933-01-0005) and control siRNA (Santa Cruz Biotechnology, sc-37007) by using Lipofectamine 3000.

### Transfections

pcDNA3.1 HA-SMYD2 WT or C13S plasmid, together with other plasmids when indicated, was transfected to H9c2 myoblasts by electroporation using Nucleofector^TM^ 2b device (Lonza). After electroporation, cells were seeded onto six-well plates at a density of 2.5 × 10^5^ cells per well for 2 days and switched to differentiation medium. SMYD2 WT or C13S mRNA was synthesized by using mMESSAGE mMACHINE T7 transcription kit (Invitrogen). Neonatal rat ventricular cardiomyocytes or HL-1 cells were transfected with SMYD2 WT or C13S mRNA using Lipofectamine MessengerMax transfection reagent (Invitrogen). HEK293 cells were transfected with HA-SMYD2 WT or C13S, and other plasmids when indicated, by using PEI-MAX.

### Cell viability assay

Differentiated H9c2 cells were treated with various stimuli, including AMA, or DMSO in a serum-free DMEM for 24 h. Alternatively, before incubation of AMA, H9c2 cells were transfected with SMYD2 siRNA, MMP-2 siRNA or control siRNA, or treated with acetyl-calpastatin (5 µM) or ARP 100 (1 µM) in serum-free DMEM for 1 h at 37 °C. Cell viability was then measured by Trypan blue assay with TC20 automated cell counter (BioRad).

### Immunofluorescence and immunostaining

After transfection of SMYD2 WT or C13S mRNA to neonatal rat ventricular cardiomyocytes, cells were incubated with AMA in DMEM (5 mM glucose) for 12 h, washed with cold PBS, and fixed with 4% paraformaldehyde or cold methanol. Fixed cells were washed three times with PBS and incubated in a permeabilization buffer (PBS with 0.1% Triton X-100) for 15 min. After incubation in a blocking buffer (PBS with 0.1% TWEEN-20 and 3% BSA) for 1 h, fixed cells were incubated with primary antibodies in a blocking buffer overnight at 4 °C. Primary antibodies include titin rabbit antibody (Novus biologicals, Cat# NBP 1-88071, α-titin-NT) (1:100 dilution), SMYD2 mouse antibody (Sigma, Cat# SAB1407760) (1:100 dilution), SMYD2 rabbit antibody (Cell Signaling, Cat# 9734) (1:100 dilution), HA-tag (6E2) mouse antibody conjugated with Alexa Fluor 488 (Cell Signaling, Cat# 2350 S) and α-actinin mouse antibody (Abcam, Cat# ab9465) (1:100 dilution). After washing with PBS, fixed cells were incubated for 1 h at room temperature with secondary antibodies, including anti-rabbit Alexa Fluor 647 secondary antibody (Invitrogen, Cat# A-21244), anti-mouse Alexa Fluor 647 secondary antibody (Invitrogen, Cat# A-21235), anti-rabbit Alexa Fluor 488 secondary antibody (Invitrogen, Cat# A-11008) and anti-mouse Alexa Fluor 488 secondary antibody (Invitrogen, Cat# A-11001). After washing with PBS, coverslips were mounted onto the microscopic plate with a DAPI-containing mounting solution and analyzed under confocal microscope. Similarly, H9c2 cells expressing HA-SMYD2 WT or C13S with either pEGFP-C1 beta-actin or pCMV-mCherry-MHC-IIA were treated with AMA, fixed, and analyzed in a similar manner. All images were captured by Zeiss LSM 780 confocal microscope with oil objectives (100 × /1.4NA, 63 × /1.4NA and 40 × /1.3NA) and individual channels for DAPI (excitation: 405 nm, emission: 410–467 nm), EGFP (excitation: 488 nm, emission: 499–552 nm), mCherry (excitation: 561 nm, emission: 572–647 nm), and Alexa 647 (excitation: 633 nm, emission: 640–721 nm).

### Analysis of myofibril orientation

All confocal microscope images were preprocessed by the Fiji software: images were subjected to sharpening and smoothing processes, and converted into 8-bit grayscale format files, which were then subjected to despeckle operation. Preprocessed images were analyzed by FiberFit software to obtain the fiber dispersion parameter (*k*) and the mean fiber orientation (*µ*):^34^ to remove noises, three sets of lower and upper cutoff frequency (50 and 250, 75 and 250, 100 and 250) were used to get three *k* values in individual images where the highest *k* value was selected for the given image. About 30 images in each experiment (triplicate, total 90 images) were processed to get the *k* values in individual conditions. The *k* value was used to quantify the degree of fiber alignment. Low *k* values represent the disordered networks, whereas high *k* values represent aligned networks.^34^

### Analysis of colocalization

Volocity 6.3.1 software was utilized to obtain the Pearson’s Correlation Coefficient (*Rr*) for colocalization between titin and SMYD2.

### Immunoblotting analyses

Differentiated H9c2 cells or HL-1 cells transfected with HA-SMYD2 WT or C13S, or appropriate siRNA were subjected to serum starvation for 12 h and treated with AMA (2 μg/mL) in DMEM (5 mM glucose) for 12 h. Alternatively, cells were treated with ARP 100 (10 µM) for 1 h before incubation with AMA. Cells were then lysed with a lysis buffer. Proteins in lysates were resolved by SDS-PAGE and transferred to PVDF membrane. The membrane was blocked and incubated with primary antibodies toward α-actinin (Abcam, Cat# ab9465) (1:500), Hsp90 (BD transduction, Cat# 610418) (1:1000), actin (Abcam, Cat# ab3280) (1:2000), HA-tag (Biolegend, Cat# 901502) (1:1000), cardiac heavy chain myosin (Abcam, ab50967) (1:200), troponin I (Cell Signaling, Cat# 4002 S) (1:1000), β-tubulin (Santa Cruz) (1:1000), SMYD2 (Cell Signaling, Cat# 9734) (1:1000), or MMP-2 (Cell Signaling, Cat#4022 S) (1:1000) diluted in a blocking buffer at 4 °C overnight. Proteins were then visualized by chemiluminescence using appropriate HRP-conjugated secondary antibodies. Supplementary Fig. 19 shows the uncropped or original blots corresponding to the panels displayed in the figures.

### Co-immunoprecipitation (co-IP) analysis

After transfection of pcDNA3.1 HA-SMYD2 WT or C13S plasmids, HEK293 cells were incubated with AMA (2 μg/mL) in glucose-free DMEM for 2 h. Cells were then lysed at 4 °C in a lysis buffer (100 mM Tris-HCl pH 7.4, NaCl 150 mM, 0.1% Tween 20 and protease inhibitor cocktail). Lysates were incubated with Hsp90 antibody (BD Bioscience, Cat# 610418) for 1 h at 4 °C and further incubated with Protein-G agarose beads (Invitrogen) for overnight at 4 °C. Proteins on beads were washed three times with TBST and eluted with an SDS-loading buffer. Eluted proteins were analyzed by Western blotting using Hsp90 antibody (BD transduction, Cat# 610418) or HA-antibody (Biolegend, Cat# 901502). Similarly, co-IP of N2A-FLAG and HA-SMYD2 was analyzed by using HA-antibody (Biolegend, Cat# 901502) and FLAG-antibody (Sigma, Cat# F1804).

### In vitro binding assay

For in vitro binding analysis between SMYD2 and Hsp90 or N2A, N-terminal GST-tagged Hsp90 or N2A was incubated with glutathione beads at 4 °C in a buffer (Tris-HCl pH 7.4, 150 mM NaCl) for 1 h. After washing beads with the same buffer to remove any unbound protein, equal amounts of SMYD2-SH and SMYD2-SSG were incubated with beads for 1 h at 4 °C. Proteins on beads were then washed with Tris-HCl (pH 7.4) buffer, eluted with an SDS-loading buffer, resolved by SDS-PAGE, and analyzed by Coomassie stains. The binding interactions between GST-N2A and oxidized forms of SMYD2 were analyzed in the same manner. Briefly, purified SMYD2 WT or SMYD2 C13S was oxidized by addition of different concentrations of H_2_O_2_ (25, 100 and 500 µM) for different time intervals (15 and 30 min) in the presence or absence of glutathione (1 mM). SMYD2 without and with oxidation were dialyzed for 30 min before addition to the binding buffer, with 20-fold dilution, containing GST-N2A and glutathione agarose.

### In vitro degradation of N2A by MMP-2 or calpain 1

Purified N2A was incubated with human recombinant active MMP-2 (Millipore) or calpain 1 (Sigma) in a buffer (Tris-HCl pH 7.4, 150 mM NaCl and 5 mM CaCl_2_) at 37 °C in the absence or presence of SMYD2 C13S (a ratio of SMYD2 to N2A was in the range between 0.5 and 5). Each reaction was quenched by addition of an SDS-loading buffer. Proteins were resolved by SDS-PAGE and visualized by Coomassie stains. To examine the N2A degradation by MMP-2 in the presence of oxidized SMYD2, purified SMYD2 WT or SMYD2 C13S was oxidized with different concentrations of H_2_O_2_, as described above, in the presence or absence of glutathione. SMYD2 without and with oxidation were dialyzed for 30 min and added to the digestion buffer with a 12-fold dilution. The reaction was initiated by addition of MMP-2 and N2A, and incubated for 2 h at 37 °C. After quenching the reaction, proteins were resolved by 10% SDS-PAGE and visualized by Coomassie stains.

### Isolation, digestion, and electrophoresis of myofibrils

Mice (C57BL/6) and rat (Brown Norway × Sprague Dawley) were used under the guidelines of protocols approved by the Wayne State University Animal Care and Use Committee. Gastrocnemius muscle of 2 or 3-month-old mice was rapidly removed after euthanasia and rinsed with ice-cold PBS. Similarly, soleus muscle and left ventricle were dissected from a female rat (193-days-old, Brown Norway × Sprague Dawley). The tissue was flash-frozen in liquid nitrogen and stored at −80 °C until use. Skeletal myofibrils were isolated from mouse gastrocnemius muscle.^66^ Initially the frozen muscle was thawed on ice and cut into small pieces, followed by homogenization in a cold lysis buffer (10 mM Tris-HCl, pH 7.0, 5 mM EGTA, 130 mM NaCl, 5 mM KCl, 1 mM MgCl_2_, 1 mM NaN_3_, 1 mM DTT, 0.1 mM PMSF, 10 μM E-64, 100 μM leupeptin and protease inhibitor cleavage cocktail) using an electronic homogenizer. Homogenates were then pelleted at 4 °C by centrifugation for 5 min at 2,500 x *g*. Pellets were then washed in a cold washing buffer (10 mM Tris-HCl, pH 7.0, 60 mM KCl, 30 mM imidazole, 2 mM MgCl_2_, 1 mM DTT, 0.1 mM PMSF, 10 μM E-64, 100 μM leupeptin and protease inhibitor cleavage cocktail) with 0.5% (v/v) Triton X-100 for once and without Triton X-100 for three times. Myofibrils were then suspended in a cold suspension buffer (20 mM MOPS, pH 7.0, 1 mM EGTA, 5 mM MgCl_2_, 100 mM KCl, 0.1 mM PMSF, 10 μM E-64, 100 μM leupeptin and protease inhibitor cleavage cocktail), filtered through a 70 µm pore size nylon mesh (sterile cell strainer) (Fisher Scientific, Cat# 22363548), and stored at 4 °C until its use. Myofibril digestion was performed according to the previous method.^67^ Briefly, freshly prepared myofibrils were washed three times using a suspension buffer without protease inhibitors. Myofibrils were incubated with human recombinant active MMP-2 (0.4 µg) in the digestion buffer (Tris-HCl pH 7.4, 150 mM NaCl, 2.0 mM CaCl_2_) at 37 °C for different incubation times (0–30 min). Alternatively, myofibrils were pre-incubated with SMYD2 (25 µg) or MMP-2 was pre-incubated with ARP-100 (100 nM) at 37 °C for 15 min before addition to the digestion buffer. The reaction was then quenched by addition of a 2X urea-loading buffer (0.05 M Tris-HCl, pH 6.8, 8 M urea, 2 M thiourea, 3% SDS, 75 mM dithiothreitol, 0.03% bromophenol blue, 25% glycerol). Proteins were then separated by 1% vertical SDS-agarose gel with the Hoefer™ SE 600 Chroma Vertical Electrophoresis System, using a lower buffer (50 mM Tris-base, 0.384 M glycine, and 0.1% SDS) and an upper buffer (a lower buffer with 10 mM 2-mercaptoethanol).^68^

### Electrophoresis of titin

To examine degradation of titin in HL-1 cells, HL-1 cells were lysed in a RIPA buffer (50 mM Tris, pH 7.4, 150 mM NaCl, 1% NP-40, 0.25% sodium deoxycholate, 1 mM EDTA, 10 μM E-64, 100 μM leupeptin, and protease inhibitor cleavage cocktail) containing DTT (50 mM) and β-mercaptoethanol (50 mM). The collected lysates were incubated at 4 °C for 45 min, and centrifuged at 21,000 *×* g for 15 min. The supernatant was collected. Proteins were then separated by 1% vertical SDS-agarose gel with the Hoefer™ SE 600 Chroma Vertical Electrophoresis System, as described above.

### Statistical analysis

Data are represented as the means ± SD or the median with 95% CI and were statistically analyzed by two-way ANOVA followed by Bonferroni’s post-hoc test or one-way ANOVA followed by Tukey’s post-hoc test, or Student’s t-test with Welch’s correction. The value *p* < 0.05 was considered as statistically significant.

### Additional methods

Further details of the methods used are described in the supplementary methods section of the Supplementary Information.

## Electronic supplementary material

Supplementary Information

## Data Availability

The mass spectrometry data have been deposited to the ProteomeXchange Consortium (http://proteomecentral.proteomexchange.org) via the PRIDE partner repository with the dataset identifier PXD011123. All other data that support the findings of this study are available from the corresponding author upon reasonable request.

## References

[1] Burgoyne JR, Mongue-Din H, Eaton P, Shah AM (2012). Redox signaling in cardiac physiology and pathology. Circ. Res..

[2] Chen YR, Zweier JL (2014). Cardiac mitochondria and reactive oxygen species generation. Circ. Res..

[3] Chouchani ET (2014). Ischaemic accumulation of succinate controls reperfusion injury through mitochondrial ROS. Nature.

[4] Smith MA, Reid MB (2006). Redox modulation of contractile function in respiratory and limb skeletal muscle. Resp. Physiol. Neurobi.

[5] Gorlach A, Bertram K, Hudecova S, Krizanova O (2015). Calcium and ROS: a mutual interplay. Redox Biol..

[6] Miura H (2003). Role for hydrogen peroxide in flow-induced dilation of human coronary arterioles. Circ. Res..

[7] Saitoh SI (2006). Hydrogen peroxide - a feed-forward dilator that couples myocardial metabolism to coronary blood flow. Arter. Throm. Vasc. Biol..

[8] Canty JM, Iyer VS (2007). Hydrogen peroxide and metabolic coronary flow regulation. J. Am. Coll. Cardiol..

[9] Kalogeris T, Bao YM, Korthuis RJ (2014). Mitochondrial reactive oxygen species: a double edged sword in ischemia/reperfusion vs preconditioning. Redox Biol..

[10] Giordano FJ (2005). Oxygen, oxidative stress, hypoxia, and heart failure. J. Clin. Invest..

[11] Tidball JG, Wehling-Henricks M (2007). The role of free radicals in the pathophysiology of muscular dystrophy. J. Appl. Physiol..

[12] Kandasamy AD, Chow AK, Ali MAM, Schulz R (2010). Matrix metalloproteinase-2 and myocardial oxidative stress injury: beyond the matrix. Cardiovasc. Res..

[13] Letavernier E (2012). The role of calpains in myocardial remodelling and heart failure. Cardiovasc. Res..

[14] Willis MS, Schisler JC, Portbury AL, Patterson C (2009). Build it up-Tear it down: protein quality control in the cardiac sarcomere. Cardiovasc. Res..

[15] Ali MAM (2010). Titin is a target of matrix metalloproteinase-2 implications in myocardial ischemia/reperfusion injury. Circulation.

[16] Linke WA (2008). Sense and stretchability: the role of titin and titin-associated proteins in myocardial stress-sensing and mechanical dysfunction. Cardiovasc. Res..

[17] Steinberg SF (2013). Oxidative Stress and Sarcomeric Proteins. Circ. Res..

[18] Beckendorf L, Linke WA (2015). Emerging importance of oxidative stress in regulating striated muscle elasticity. J. Muscle Res. Cell Motil..

[19] Mieyal JJ, Gallogly MM, Qanungo S, Sabens EA, Shelton MD (2008). Molecular mechanisms and clinical implications of reversible protein S-glutathionylation. Antioxid. Redox Signal..

[20] Samarasinghe KT, Munkanatta Godage DN, VanHecke GC, Ahn YH (2014). Metabolic synthesis of clickable glutathione for chemoselective detection of glutathionylation. J. Am. Chem. Soc..

[21] Kekulandara DN, Samarasinghe KTG, Godage DNPM, Ahn YH (2016). Clickable glutathione using tetrazine-alkene bioorthogonal chemistry for detecting protein glutathionylation. Org. Biomol. Chem..

[22] Samarasinghe KTG (2016). A clickable glutathione approach for identification of protein glutathionylation in response to glucose metabolism. Mol. Biosyst..

[23] Spellmon N, Holcomb J, Trescott L, Sirinupong N, Yang Z (2015). Structure and function of SET and MYND domain-containing proteins. Int. J. Mol. Sci..

[24] Huang J (2006). Repression of p53 activity by Smyd2-mediated methylation. Nature.

[25] Cho HS (2012). RB1 Methylation by SMYD2 enhances cell cycle progression through an increase of RB1 phosphorylation. Neoplasia.

[26] Zhang X (2013). Regulation of estrogen receptor a by histone methyltransferase SMYD2-mediated protein methylation. Proc. . Natl. Acad. Sci. U. S. A..

[27] Hamamoto R, Toyokawa G, Nakakido M, Ueda K, Nakamura Y (2014). SMYD2-dependent HSP90 methylation promotes cancer cell proliferation by regulating the chaperone complex formation. Cancer Lett..

[28] Olsen JB (2016). Quantitative profiling of the activity of protein lysine methyltransferase SMYD2 Using SILAC-based proteomics. Mol. Cell. Proteom..

[29] Du SJ, Tan XG, Zhang JS (2014). SMYD proteins: key regulators in skeletal and cardiac muscle development and function. Anat. Rec..

[30] Voelkel T (2013). Lysine methyltransferase Smyd2 regulates Hsp90-mediated protection of the sarcomeric titin springs and cardiac function. Biochim. Biophys. Acta.

[31] Donlin LT (2012). Smyd2 controls cytoplasmic lysine methylation of Hsp90 and myofilament organization. Gene Dev..

[32] Xu ST, Zhong C, Zhang TL, Ding JP (2011). Structure of human lysine methyltransferase Smyd2 reveals insights into the substrate divergence in Smyd proteins. J. Mol. Cell Biol..

[33] Diehl F (2010). Cardiac deletion of Smyd2 is dispensable for mouse heart development. PLoS. One..

[34] Morrill EE (2016). A validated software application to measure fiber organization in soft tissue. Biomech. Model. Mechanobiol..

[35] Cazorla O (2000). Differential expression of cardiac titin isoforms and modulation of cellular stiffness. Circ. Res..

[36] Kojic S, Radojkovic D, Faulkner G (2011). Muscle ankyrin repeat proteins: their role in striated muscle function in health and disease. Crit. Rev. Clin. Lab. Sci..

[37] Lovett DH (2012). A Novel intracellular isoform of matrix metalloproteinase-2 induced by oxidative stress activates innate immunity. PLoS. One..

[38] Wang L (2011). Structure of human SMYD2 protein reveals the basis of p53 tumor suppressor methylation. J. Biol. Chem..

[39] Xu G (2015). The histone methyltransferase Smyd2 is a negative regulator of macrophage activation by suppressing interleukin 6 (IL-6) and tumor necrosis factor alpha (TNF-alpha) production. J. Biol. Chem..

[40] Paulsen CE, Carroll KS (2013). Cysteine-mediated redox signaling: chemistry, biology, and tools for discovery. Chem. Rev..

[41] Miyata Y (2012). Cysteine reactivity distinguishes redox sensing by the heat-inducible and constitutive forms of heat shock protein 70. Chem. Biol..

[42] Marinelli P (2018). A single cysteine post-translational oxidation suffices to compromise globular proteins kinetic stability and promote amyloid formation. Redox Biol..

[43] Grutzner A (2009). Modulation of titin-based stiffness by disulfide bonding in the cardiac titin N2-B unique sequence. Biophys. J..

[44] Kumar S, Ratnikov BI, Kazanov MD, Smith JW, Cieplak P (2015). CleavPredict: a Platform for Reasoning about Matrix Metalloproteinases Proteolytic Events (vol 10, e0127877, 2015). PLoS. One..

[45] Liu ZX (2011). GPS-CCD: A Novel Computational Program for the Prediction of Calpain Cleavage Sites. PLoS. One..

[46] Ulanova Anna, Gritsyna Yulia, Vikhlyantsev Ivan, Salmov Nikolay, Bobylev Alexander, Abdusalamova Zarema, Rogachevsky Vadim, Shenkman Boris, Podlubnaya Zoya (2015). Isoform Composition and Gene Expression of Thick and Thin Filament Proteins in Striated Muscles of Mice after 30-Day Space Flight. BioMed Research International.

[47] Chouchani ET (2016). A Unifying Mechanism for Mitochondrial Superoxide Production during Ischemia-Reperfusion Injury. Cell. Metab..

[48] Kusuoka H, Porterfield JK, Weisman HF, Weisfeldt ML, Marban E (1987). Pathophysiology and Pathogenesis of Stunned Myocardium - Depressed Ca-2+Activation of Contraction as a Consequence of Reperfusion-Induced Cellular Calcium Overload in Ferret Hearts. J. Clin. Invest..

[49] Macfarlane NG, Miller DJ (1992). Depression of Peak Force without Altering Calcium Sensitivity by the Superoxide Anion in Chemically Skinned Cardiac-Muscle of Rat. Circ. Res..

[50] Ferdinandy P, Danial H, Ambrus I, Rothery RA, Schulz R (2000). Peroxynitrite is a major contributor to cytokine-induced myocardial contractile failure. Circ. Res..

[51] Canton M, Neverova I, Menabo R, Van Eyk J, Di Lisa F (2004). Evidence of myofibrillar protein oxidation induced by postischemic reperfusion in isolated rat hearts. Am. J. Physiol. Heart Circ. Physiol..

[52] Chen FC, Ogut O (2006). Decline of contractility during ischemia-reperfusion injury: actin glutathionylation and its effect on allosteric interaction with tropomyosin. Am. J. Physiol. Cell. Physiol..

[53] Polewicz D (2011). Ischemia induced peroxynitrite dependent modifications of cardiomyocyte MLC1 increases its degradation by MMP-2 leading to contractile dysfunction. J. Cell. Mol. Med..

[54] Avner BS (2012). Myocardial infarction in mice alters sarcomeric function via post-translational protein modification. Mol. Cell. Biochem..

[55] Pan KT (2014). Mass spectrometry-based quantitative proteomics for dissecting multiplexed redox cysteine modifications in nitric oxide-protected cardiomyocyte under hypoxia. Antioxid. Redox Signal..

[56] Mollica JP (2012). S-Glutathionylation of troponin I (fast) increases contractile apparatus Ca2+sensitivity in fast-twitch muscle fibres of rats and humans. J. Physiol..

[57] Alegre-Cebollada J (2014). S-Glutathionylation of cryptic cysteines enhances titin elasticity by blocking protein folding. Cell.

[58] Samarasinghe KTG, Ahn YH (2015). Synthesizing clickable glutathione by glutathione synthetase mutant for detecting protein glutathionylation. Synlett.

[59] Castro JP, Jung T, Grune T, Siems W (2017). 4-Hydroxynonenal (HNE) modified proteins in metabolic diseases. Free Radic. Biol. Med..

[60] Ternette N (2013). Inhibition of mitochondrial aconitase by succination in fumarate hydratase deficiency. Cell Rep..

[61] Hein S (2009). Deposition of nonsarcomeric alpha-actinin in cardiomyocytes from patients with dilated cardiomyopathy or chronic pressure overload. Exp. Clin., Cardiol..

[62] Dispersyn GD, Geuens E, Donck LV, Ramaekers FCS, Borgers M (2001). Adult rabbit cardiomyocytes undergo hibernation-like dedifferentiation when co-cultured with cardiac fibroblasts. Cardiovasc. Res..

[63] Bell RAV, Al-Khalaf M, Megeney LA (2016). The beneficial role of proteolysis in skeletal muscle growth and stress adaptation (vol 6, 16, 2016). Skelet. Muscle.

[64] Bullard B (2004). Association of the chaperone alpha B-crystallin with titin in heart muscle. J. Biol. Chem..

[65] Nesvizhskii AI, Keller A, Kolker E, Aebersold R (2003). A statistical model for identifying proteins by tandem mass spectrometry. Anal. Chem..

[66] Gunther LK (2016). Effect of N-Terminal Extension of Cardiac Troponin I on the Ca2+Regulation of ATP Binding and ADP Dissociation of Myosin II in Native Cardiac Myofibrils. Biochemistry.

[67] Zhang ZL, Biesiadecki BJ, Jin JP (2006). Selective deletion of the NH2-terminal variable region of cardiac troponin T in ischemia reperfusion by myofibril-associated mu-calpain cleavage. Biochemistry.

[68] Chung CS (2013). Shortening of the elastic tandem immunoglobulin segment of titin leads to diastolic dysfunction. Circulation.

