# Supplementary material for: SMYD2 glutathionylation contributes to degradation of sarcomeric proteins
Source: Nat Commun. 2018 Oct 18;9:4341. doi: 10.1038/s41467-018-06786-x (PMC6194001; doi:10.1038/s41467-018-06786-x)
Supplement: Supplementary file 1 — Supplementary Information [file 41467_2018_6786_MOESM1_ESM.pdf]

# Supplementary Information

## SMYD2 Glutathionylation Contributes to Degradation of Sarcomeric Proteins

Munkanatta Godage *et al*.

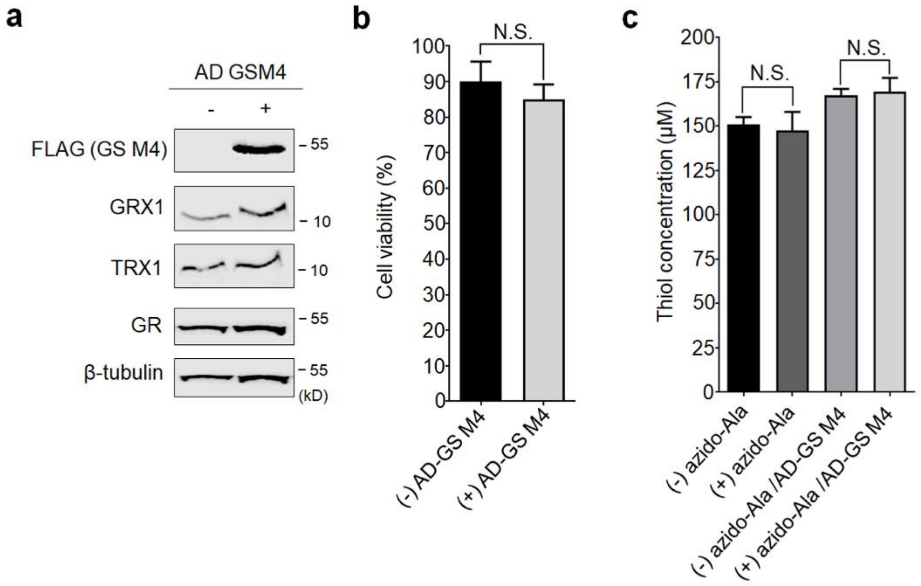


**Supplementary Figure 1. A clickable glutathione approach does not induce significant cell toxicity or alteration of redox systems**. Differentiated H9c2 cells were incubated with adenovirus expressing a glutathione synthetase mutant (GS M4) for 48 h. (**a**) Level of GS M4 and other redox enzymes. Lysates were analyzed by Western blotting. Blots are the representative of at least 3 independent experiments. (**b**) Cell viability after expression of GS M4. Collected cells were analyzed for viability by Trypan blue assay. (**c**) A total level of thiols in cells after expression of GS M4 with incubation of azido-Ala. Cells were lysed by freeze-thawing, and protein-free lysates were analyzed for thiol-concentrations by a bromobimane assay. Data represent the mean ± SD, n= 3 independent experiments. Difference is significant by two-tailed Student's unpaired t-test with Welch's correction (**b**) or one-way ANOVA followed by Tukey's *post-hoc* test (**c**), *p < 0.05 and N.S.= non-significant.

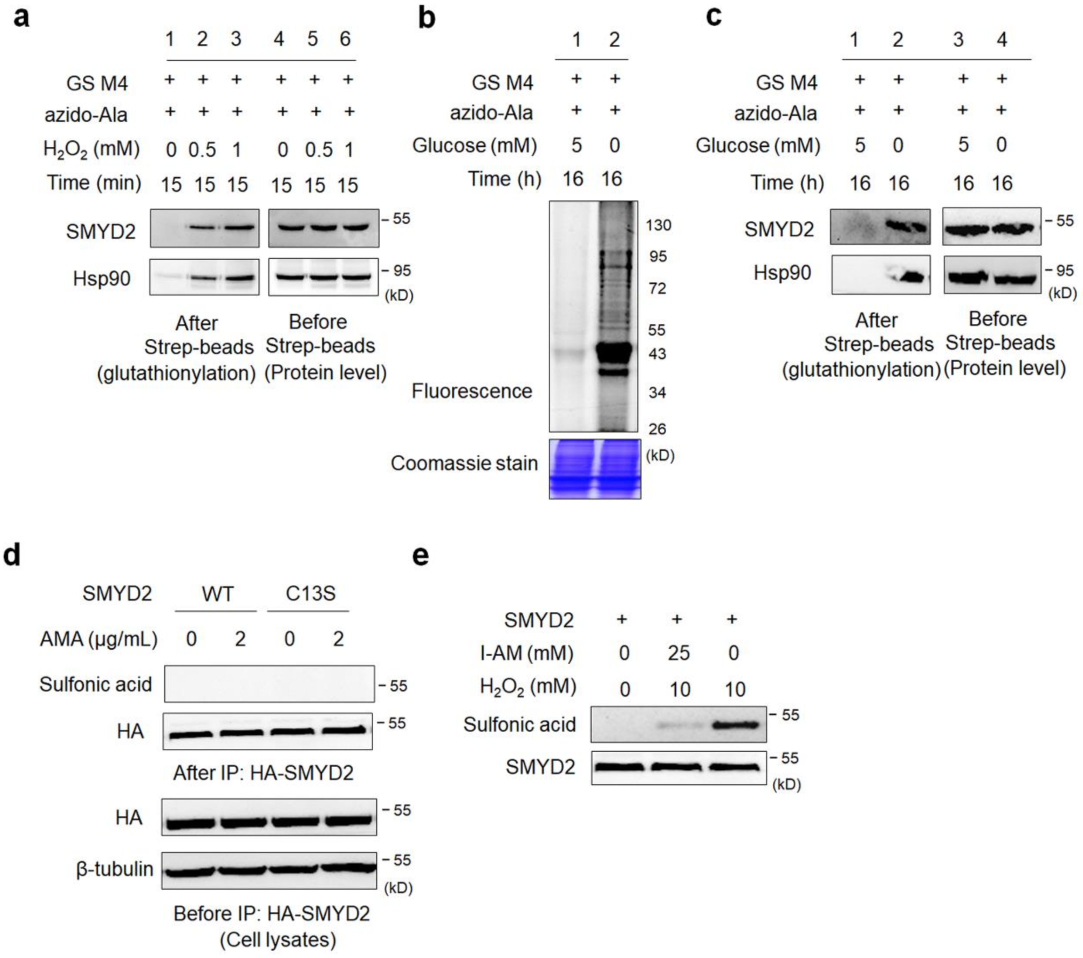


**Supplementary Figure 2. Detection of SMYD2 glutathionylation in differentiated H9c2 cells**. (**a**-**c**) Glutathione synthetase mutant (GS M4) was expressed in differentiated H9c2 myocytes. After incubation of azido-Ala, cells were subjected to $H_2O_2$ (**a**) or glucose deprivation (**b-c**). Collected lysates were then subjected to click reaction with rhodamine-alkyne or biotin-alkyne. Glutathionylated proteins were analyzed by Western blotting with individual antibodies before and after pull-downs with streptavidin-agarose. (**d**) Analysis of SMYD2 oxidations with antibody that detects the sulfonic acid group. H9c2 cells expressing SMYD2 WT or C13S were treated with antimycin A (AMA), and the sulfonic acid formation in SMYD2 was probed by Western blotting after pull-down of SMYD2. (**e**) Validation of the sulfonic acid-antibody for detection of sulfonic acid. To purified SMYD2 was added $H_2O_2$ (10 mM) without or with pre-treatment of iodoacetamide (I-AM) that blocks Cys residues.  Blots are the representative of at least 3 independent experiments.

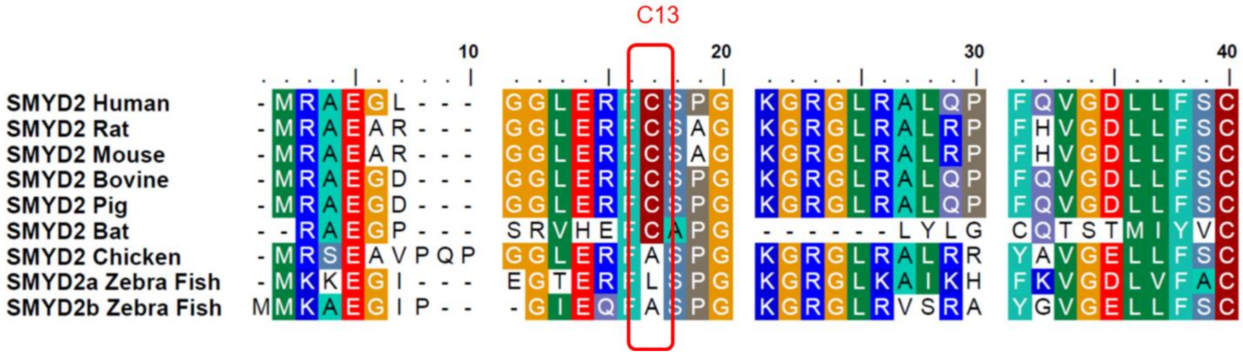


**Supplementary Figure 3. The sequence alignment of SMYD2 Cys 13 in SMYD2 orthologs.**

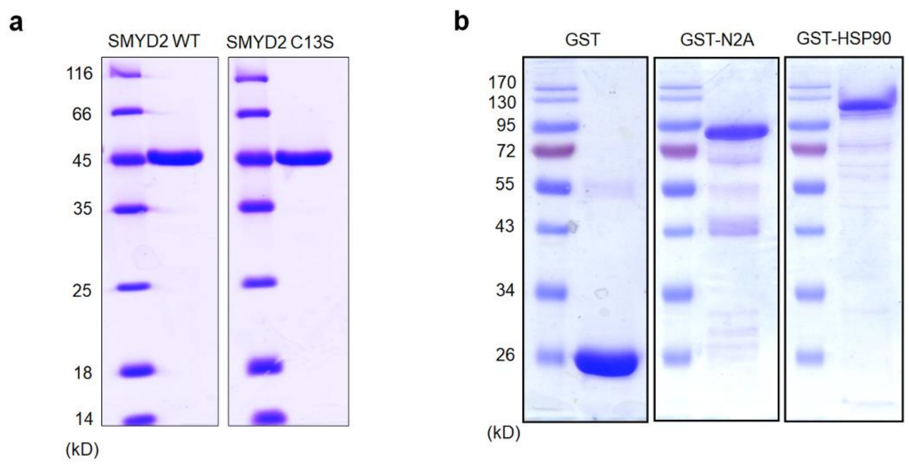


**Supplementary Figure 4. Purity of recombinant proteins**. (**a**) SMYD2 WT and C13S were expressed in *E. co*li and purified by affinity-columns and ion-exchange columns. (**b**) GST, GST-N2A, and GST-Hsp90 were expressed in *E. coli* and purified by affinity-column (glutathione-agarose).

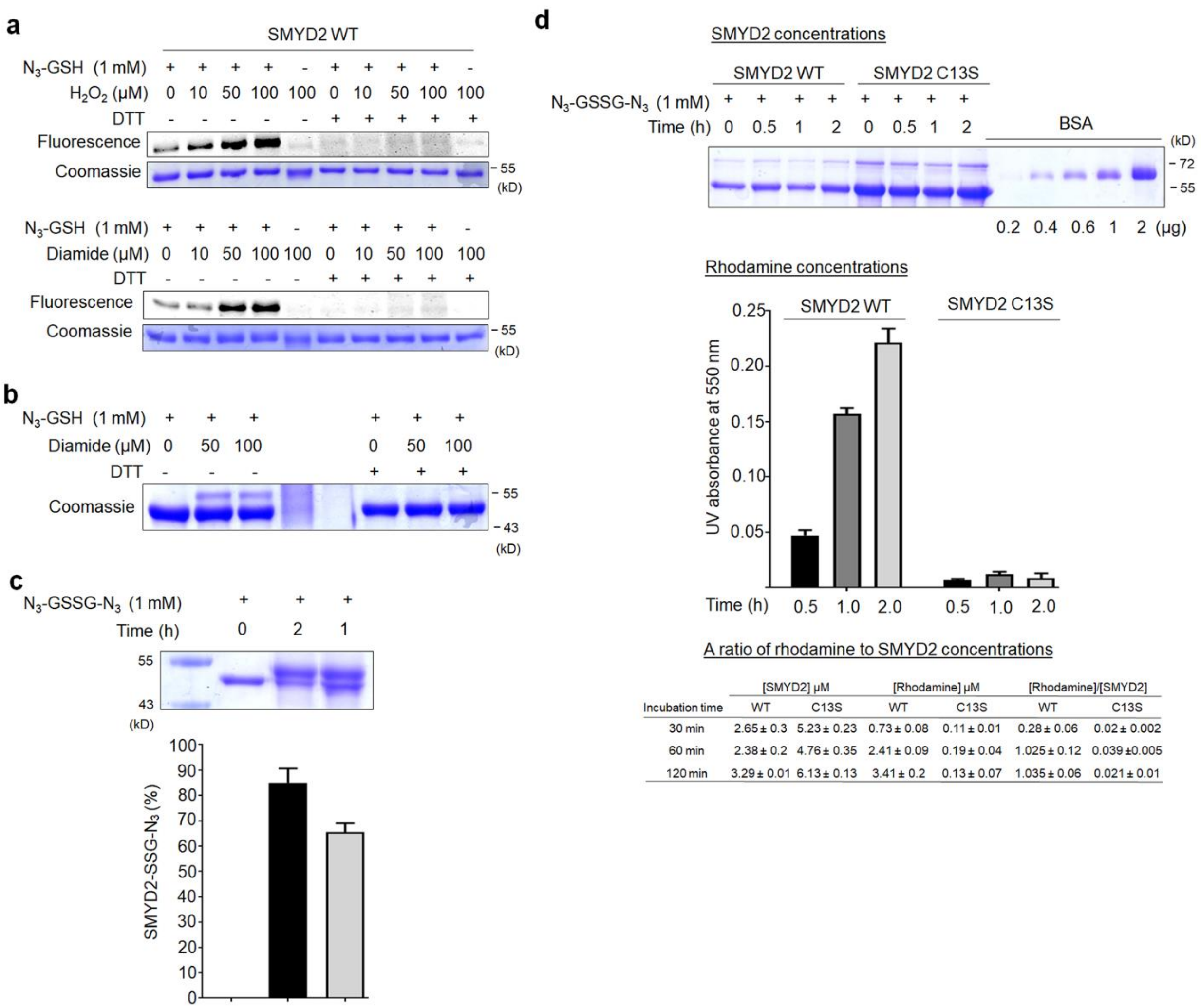


| | [SMYD2] μM | | [Rhodamine] μM | | [Rhodamine]/[SMYD2] | |
|---|---|---|---|---|---|---|
| Incubation time | WT | C13S | WT | C13S | WT | C13S |
| 30 min | 2.65 ± 0.3 | 5.23 ± 0.23 | 0.73 ± 0.08 | 0.11 ± 0.01 | 0.28 ± 0.06 | 0.02 ± 0.002 |
| 60 min | 2.38 ± 0.2 | 4.76 ± 0.35 | 2.41 ± 0.09 | 0.19 ± 0.04 | 1.025 ± 0.12 | 0.039 ±0.005 |
| 120 min | 3.29 ± 0.01 | 6.13 ± 0.13 | 3.41 ± 0.2 | 0.13 ± 0.07 | 1.035 ± 0.06 | 0.021 ± 0.01 |

**Supplementary Figure 5. In-gel analysis of SMYD2 glutathionylation with azido-glutathione *in vitro*.** (**a-b**) Detection of SMYD2 glutathionylation. Purified SMYD2 WT was incubated with azido-glutathione. $H_2O_2$ or diamide was then added for 5 min at room temperature. After treating iodoacetamide (25 mM) for 15 min, proteins were precipitated, re-dissolved in PBS, conjugated with rhodamine-alkyne (**a**) or 2-kD PEG-alkyne (**b**), and run on a gel for fluorescence detection or Coomassie stain. Blots are representatives from at least 3 independent experiments. (**c-d**) Measurement of the level of SMYD2 glutathionylation. Purified SMYD2 WT or C13S was incubated with oxidized azido-glutathione ($N_3$-GSSG-$N_3$). At indicated time points, proteins were blocked with iodoacetamide. After click-reaction with 2-kD PEG-alkyne (**c**) or rhodamine-alkyne (**d**), proteins were precipitated to remove any remaining alkyne-derivatives and re-dissolved in

PBS. Proteins in PBS were analyzed to measure rhodamine absorbance intensity at 550 nm to quantify the rhodamine concentration in comparison with a calibration curve. SMYD2 protein concentration was measured by gel analysis (ImageJ software to quantify the intensity of Coomassie bands) in comparison to the BSA calibration curve. Data represent the mean ± SD, *n* = 3 independent experiments.

**a**

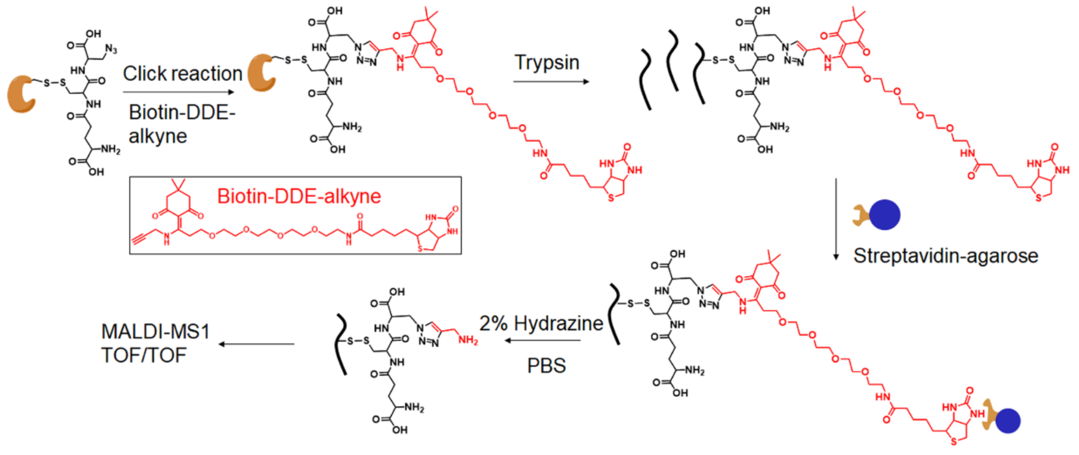


**b**

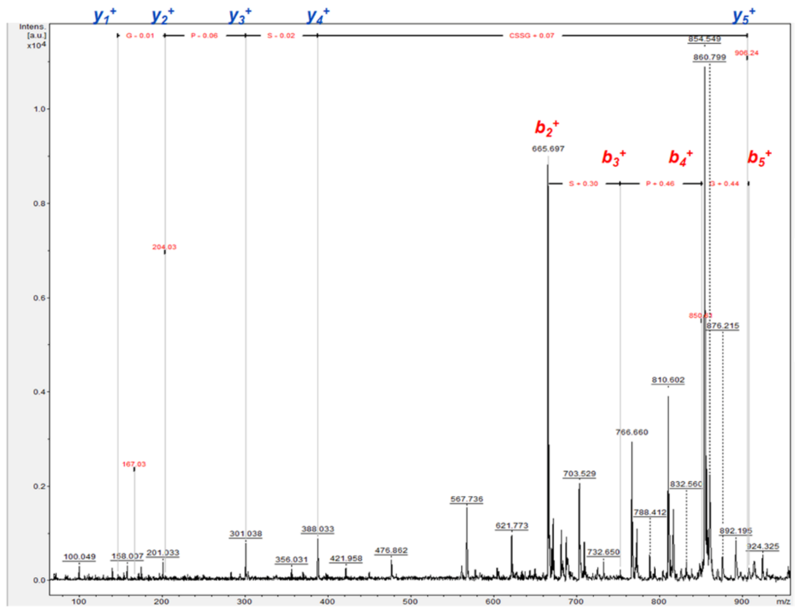

| #1 | $b^+$ | Seq. | $y^+$ | #2 |
|---|---|---|---|---|
| 1 | 148.0757 | F | | 6 |
| 2 | 666.0849 | C* | 906.2282 | 5 |
| 3 | 753.1169 | S | 388.2191 | 4 |
| 4 | 850.1697 | P | 301.187 | 3 |
| 5 | 907.1911 | G | 204.1343 | 2 |
| 6 | | K | 147.1128 | 1 |

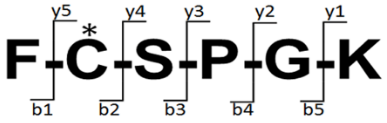

**c**

AEGLGGLERF**C**$_{13}$*SPGK

| C | C Ions | C+2H | AA | Z Ions | Z+2H | Z |
|---|---|---|---|---|---|---|
| 1 | 89.1 | 44.5 | A | 1,919.9 | 960.4 | 15 |
| 2 | 218.1 | 109.1 | E | 1,848.8 | 924.9 | 14 |
| 3 | 275.1 | 137.6 | G | 1,719.8 | 860.4 | 13 |
| 4 | 388.2 | 194.1 | L | 1,662.8 | 831.9 | 12 |
| 5 | 445.2 | 222.6 | G | 1,549.7 | 775.3 | 11 |
| 6 | 502.3 | 251.1 | G | 1,492.7 | 746.8 | 10 |
| 7 | 615.3 | 307.7 | L | 1,435.6 | 718.3 | 9 |
| 8 | 744.4 | 372.2 | E | 1,322.5 | 661.8 | 8 |
| 9 | 900.5 | 450.2 | R | 1,193.5 | 597.3 | 7 |
| 10 | 1,047.6 | 523.8 | F | 1,037.4 | 519.2 | 6 |
| 11 | 1,565.7 | 782.8 | C+415 | 890.3 | 445.7 | 5 |
| 12 | 1,652.7 | 826.4 | S | 372.2 | 186.6 | 4 |
| 13 | 1,749.8 | 874.9 | P | 285.2 | 143.1 | 3 |
| 14 | 1,806.8 | 903.4 | G | 188.1 | 94.6 | 2 |
| 15 | 1,952.9 | 976.5 | K | 131.1 | 66.1 | 1 |

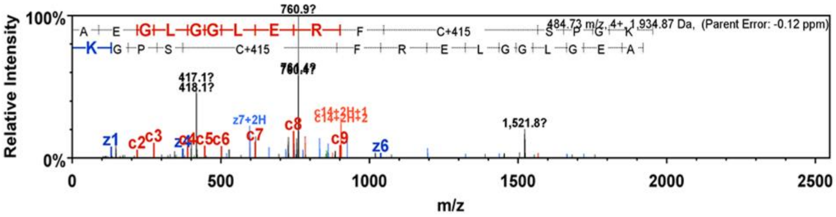


SPSELLEI**C**$_{321}$*ELS

| C | C Ions | C+2H | AA | Z Ions | Z+2H | Z |
|---|---|---|---|---|---|---|
| 1 | 105.1 | 52.5 | S | 2,103.9 | 1,052.5 | 15 |
| 2 | 202.1 | 101.1 | P | 2,016.9 | 1,009.0 | 14 |
| 3 | 289.2 | 144.6 | S | 1,919.9 | 960.4 | 13 |
| 4 | 418.2 | 209.1 | E | 1,832.8 | 916.9 | 12 |
| 5 | 531.3 | 265.6 | L | 1,703.8 | 852.4 | 11 |
| 6 | 644.4 | 322.2 | L | 1,590.7 | 795.9 | 10 |
| 7 | 773.4 | 386.7 | E | 1,477.6 | 739.3 | 9 |
| 8 | 886.5 | 443.2 | I | 1,348.6 | 674.8 | 8 |
| 9 | 1,404.6 | 702.3 | C+415 | 1,235.5 | 618.2 | 7 |
| 10 | 1,533.7 | 766.8 | E | 717.4 | 359.2 | 6 |
| 11 | 1,646.8 | 823.4 | L | 588.3 | 294.7 | 5 |
| 12 | 1,733.8 | 866.9 | S | 475.2 | 238.1 | 4 |
| 13 | 1,861.8 | 930.9 | Q | 388.2 | 194.6 | 3 |
| 14 | 1,990.9 | 995.4 | E | 260.1 | 130.6 | 2 |
| 15 | 2,137.0 | 1,068.5 | K | 131.1 | 66.1 | 1 |

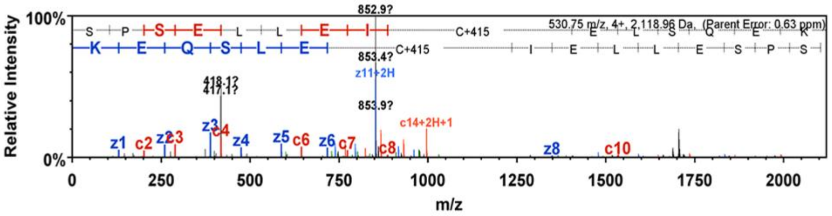


QAFY**C**$_{74}$*NVECQKEDWPMHK

| C | C Ions | C+2H | AA | Z Ions | Z+2H | Z |
|---|---|---|---|---|---|---|
| 1 | 146.1 | 73.0 | Q | 2,655.1 | 1,328.0 | 18 |
| 2 | 217.1 | 108.6 | A | 2,527.0 | 1,264.0 | 17 |
| 3 | 364.2 | 182.1 | F | 2,456.0 | 1,228.5 | 16 |
| 4 | 527.3 | 263.6 | Y | 2,308.9 | 1,155.0 | 15 |
| 5 | 1,045.4 | 522.7 | C+415 | 2,145.8 | 1,073.4 | 14 |
| 6 | 1,159.4 | 579.7 | N | 1,627.7 | 814.4 | 13 |
| 7 | 1,258.5 | 629.3 | V | 1,513.7 | 757.3 | 12 |
| 8 | 1,387.6 | 693.8 | E | 1,414.6 | 707.8 | 11 |
| 9 | 1,490.6 | 745.3 | C | 1,285.6 | 643.3 | 10 |
| 10 | 1,618.6 | 809.3 | Q | 1,182.5 | 591.8 | 9 |
| 11 | 1,746.7 | 873.4 | K | 1,054.5 | 527.7 | 8 |
| 12 | 1,875.8 | 937.9 | E | 926.4 | 463.7 | 7 |
| 13 | 1,990.8 | 995.4 | D | 797.4 | 399.2 | 6 |
| 14 | 2,176.9 | 1,088.4 | W | 682.3 | 341.7 | 5 |
| 15 | 2,273.9 | 1,137.0 | P | 496.2 | 248.6 | 4 |
| 16 | 2,405.0 | 1,202.5 | M | 399.2 | 200.1 | 3 |
| 17 | 2,542.0 | 1,271.0 | H | 268.2 | 134.6 | 2 |
| 18 | 2,688.1 | 1,344.1 | K | 131.1 | 66.1 | 1 |

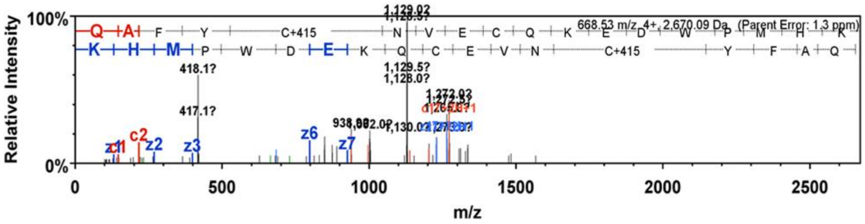


**Supplementary Figure 6. Mass analysis of glutathionylated SMYD2.** (**a**) A scheme for mass identification of glutathionylated peptides in SMYD2. SMYD2 glutathionylated by azido-glutathione was subjected to click reaction with biotin-DDE-alkyne (click chemistry tools, Inc.), which is cleavable by hydrazine, and digested by trypsin at 37°C overnight. Digested peptides were incubated with streptavidin-agarose for 3 h, and bound peptides were eluted by 2% hydrazine in PBS (pH 7.4) for 30 min (twice). The collected sample was ziptiped and analyzed for MALDI-TOF and TOF/TOF. (**b**) The spectrum of MALDI-TOF/TOF with identified ions, and the predicted mass table for individual ions. Identified *y* and *b* ions are highlighted by red and blue colors. The modification on Cys was calculated as 415.14, which adds the mass of azido-glutathione and a fragment cleaved from biotin-DDE-alkyne. (**c**) LC-MS/MS (ESI) analysis of glutathionylated SMYD2. Glutathionylated SMYD2 was analyzed by ESI LC-MS/MS after the

same procedure described in (**a**), except that glutathionylation was induced by addition of diamide (100 µM) in the presence of azido-glutathione, detecting the peptides glutathionylated at Cys13, Cys74, and Cys321.

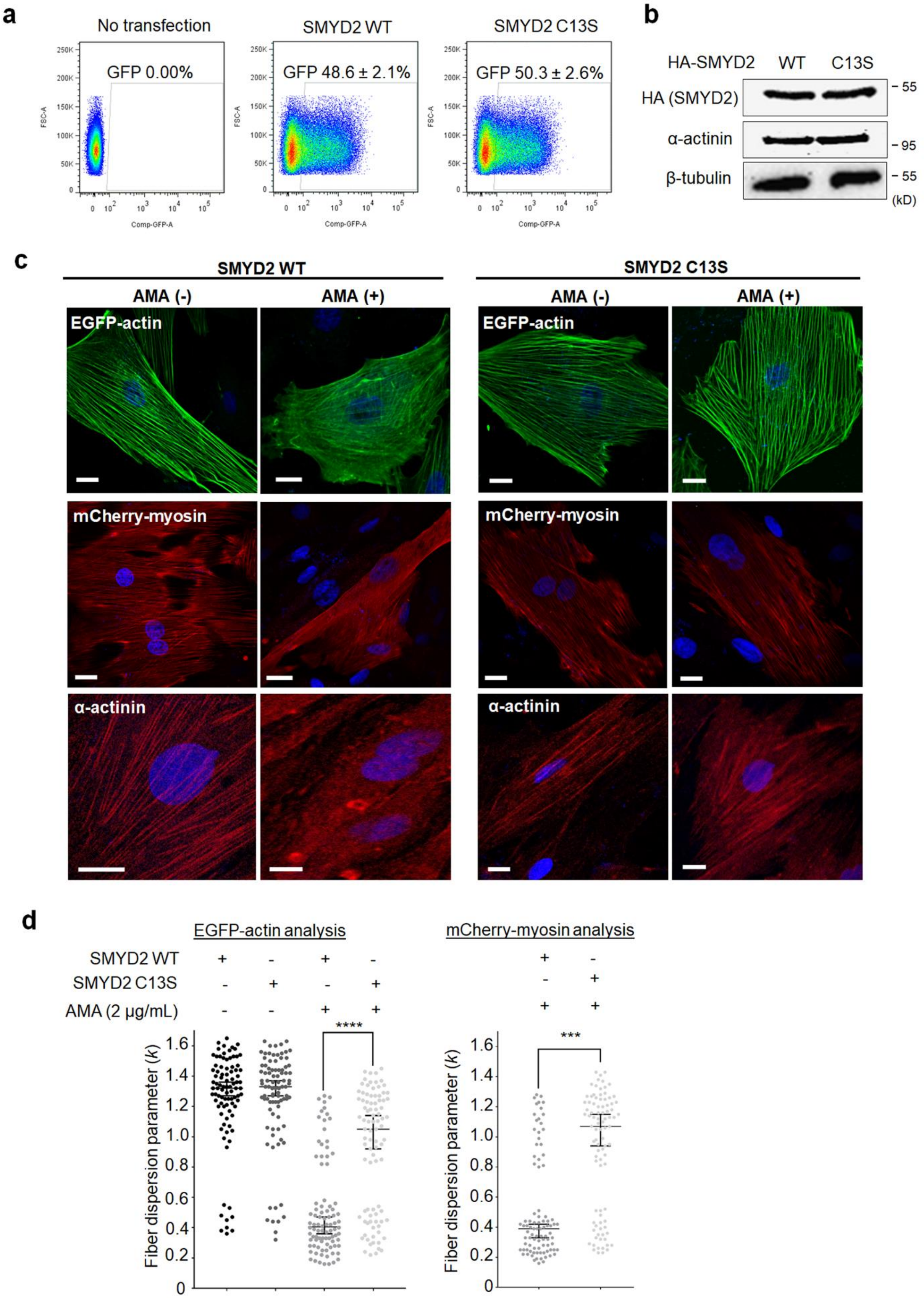
a
No transfection
GFP 0.00%
SMYD2 WT
GFP 48.6 ± 2.1%
SMYD2 C13S
GFP 50.3 ± 2.6%
FSC-A
Comp-GFP-A
b
HA-SMYD2
WT
C13S
HA (SMYD2)
α-actinin
β-tubulin
55
95
55
(kD)
c
SMYD2 WT
SMYD2 C13S
AMA (-)
AMA (+)
EGFP-actin
mCherry-myosin
α-actinin
d
EGFP-actin analysis
mCherry-myosin analysis
SMYD2 WT
SMYD2 C13S
AMA (2 µg/mL)
Fiber dispersion parameter (k)
****
***

**Supplementary Figure 7. Fluorescence imaging of sarcomeric proteins in differentiated H9c2 cells expressing SMYD2 WT or C13S.** (**a**) FACS analysis to estimate transfection efficiency of SMYD2 expression. After electroporation of EGFP-SMYD2 WT or C13S to H9c2 cells, cells were differentiated. Cells were then collected and analyzed for EGFP signals. Data represent the mean ± SD, $n = 3$ independent experiments. (**b**) Protein levels of SMYD2 and α-actinin in H9c2 myocytes after differentiation. SMYD2 WT or C13S plasmids were electroporated to H9c2 myoblasts. Cells were then differentiated for 5 days. After differentiation, cells were lysed for comparing the protein levels by Western blotting. (**c**) Fluorescence imaging or immunostaining of sarcomeric proteins in differentiated H9c2 cells. After co-expression of SMYD2 WT or C13S with EGFP-actin or mCherry-myosin to H9c2 cells, cells were differentiated. Cells were then treated with AMA for 12 h. H9c2 cells expressing SMYD2 WT or C13S were imaged by EGFP-actin, mCherry-myosin, or α-actinin immunostaining. (**d**) The alignment analysis of labelled or stained proteins with the fiber dispersion parameter ($k$) values. About 30 cells (triplicate, total 90 cells) were photographed and examined for directionality of labeled or stained proteins by using FiberFit software.[1] The median values with 95% CI are shown, $n = 3$ independent experiments. Difference is significant by one-way ANOVA followed by Tukey's *post-hoc* test (left panel) or two-tailed Student's unpaired t-test with Welch's correction (right panel), *$p < 0.05$, **$p < 0.01$, ***$p < 0.001$, ****$p < 0.0001$. Scale bars, 20 µm.

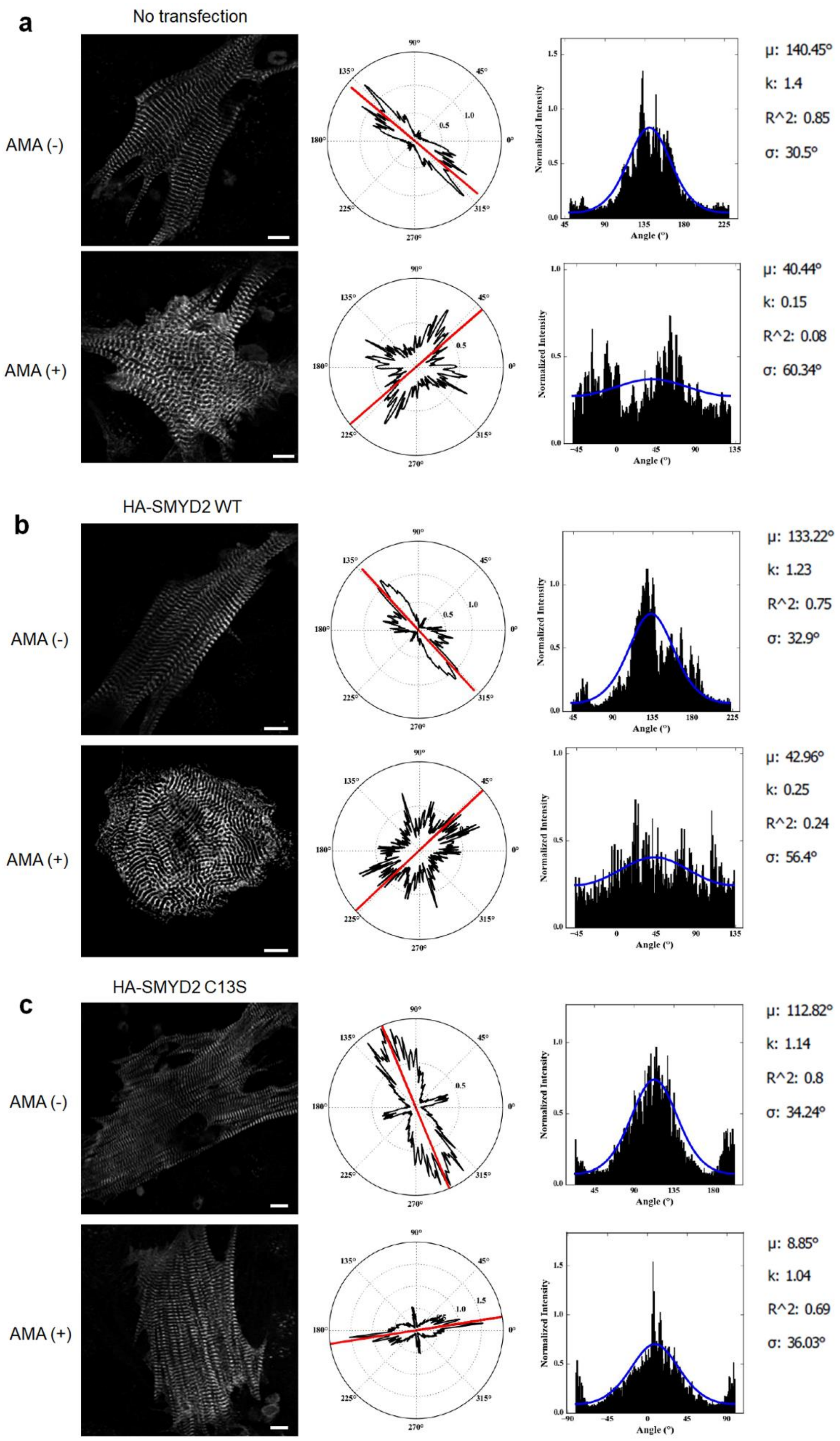
a
No transfection
AMA (-)
μ: 140.45°
k: 1.4
R^2: 0.85
σ: 30.5°
AMA (+)
μ: 40.44°
k: 0.15
R^2: 0.08
σ: 60.34°
b
HA-SMYD2 WT
AMA (-)
μ: 133.22°
k: 1.23
R^2: 0.75
σ: 32.9°
AMA (+)
μ: 42.96°
k: 0.25
R^2: 0.24
σ: 56.4°
c
HA-SMYD2 C13S
AMA (-)
μ: 112.82°
k: 1.14
R^2: 0.8
σ: 34.24°
AMA (+)
μ: 8.85°
k: 1.04
R^2: 0.69
σ: 36.03°
Normalized Intensity
Angle (°)

**Supplementary Figure 8. Analyses of myofibril directionality.** Rat neonatal cardiomyocytes without expression of SMYD2 (**a**), or with expression of SMYD2 WT (**b**) or C13S (**c**) were treated with AMA for 12 h. Individual cells were photographed and analyzed for the degree of fiber alignment (*k* value, fiber dispersion parameter) by using FiberFit software: low *k* values represent the disordered network whereas high *k* values represent aligned network.[1] Images are representatives of cells in individual conditions. The distribution of *k* values in individual conditions is shown in Figure 4c. $n = 3$ independent experiments (analyses of 30 cells from each experiment).

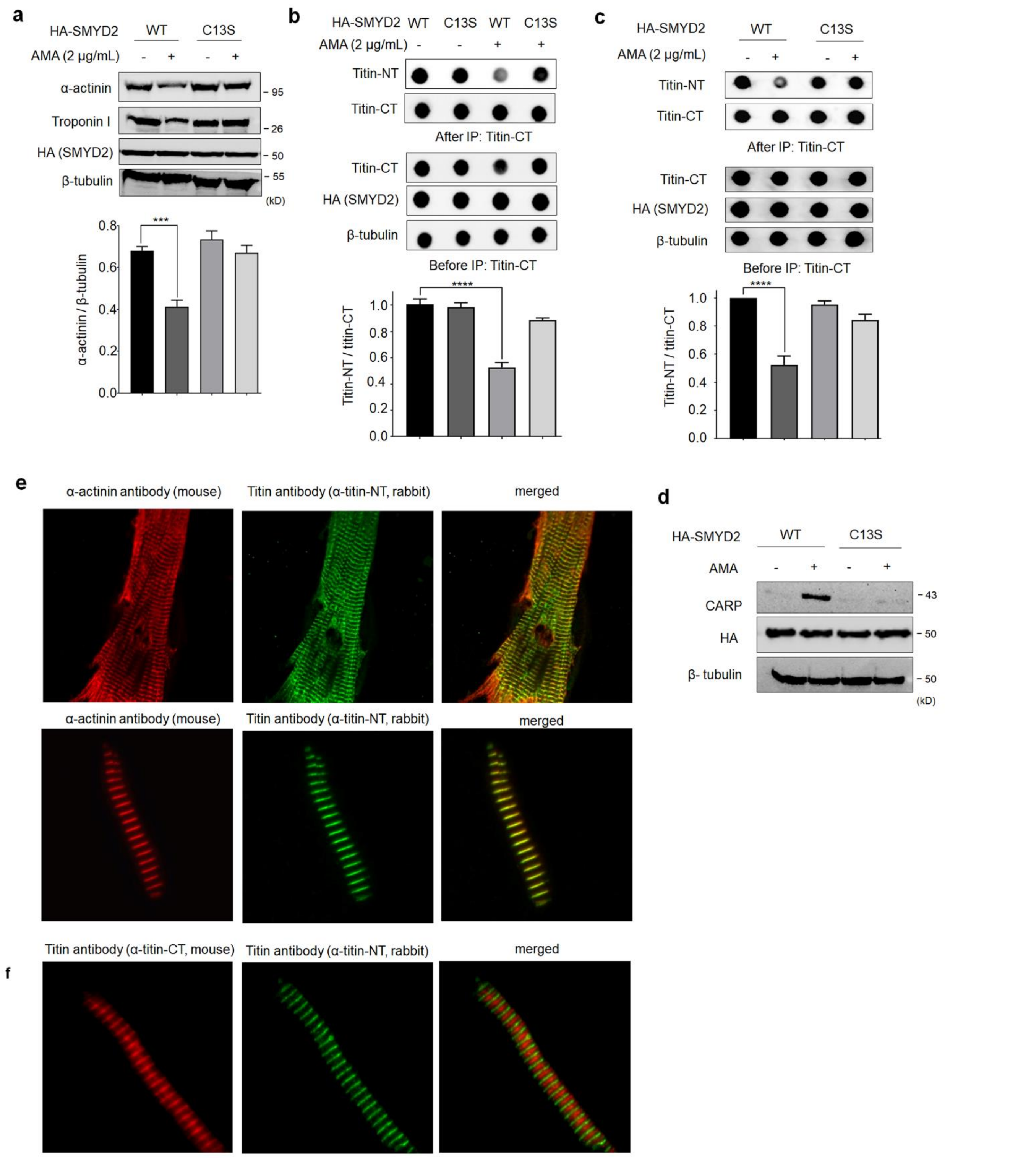


**Supplementary Figure 9. Analysis of the level of sarcomere-associated proteins in cells expressing SMYD2 WT or C13S in response to AMA.** (**a**) Sarcomeric protein levels in HL-1 cells in response to AMA. HL-1 cells expressing SMYD2 WT or C13S were treated with AMA (2

μg/mL) for 12 h. Lysates were analyzed by Western blotting. (**b-c**) Dot blot analysis of titin in HL-1 cells (**b**) or differentiated H9c2 cells (**c**) in response to AMA. To monitor the potential cleavage or degradation of titin, titin was pull-downed from lysates with antibody that binds to a C-terminal region of titin (α-titin-CT), followed by Western blotting with antibody that binds to an N-terminal region of titin (α-titin-NT). Data represent the mean ± SD, $n = 2$ independent experiments. Difference is significant by one-way ANOVA followed by Tukey's *post-hoc* test, $*p < 0.05$, $**p < 0.01$, $***p < 0.001$, $****p < 0.0001$. (**d**) The level of CARP in differentiated H9c2 cells in response to AMA. After incubation of AMA for 12 h, lysates were analyzed by Western blotting. (**e**) Validation of titin antibody (α-Titin-NT). α-Titin-NT antibody (Novus biologicals, Cat# NBP 1-88071) recognizes the N-terminus of an I-band region (Ig7-8) of titin. Immunostaining of rat neonatal cardiomyocytes (top) or isolated myofibrils (from mouse gastrocnemius) (bottom) with α-titin-NT antibody shows a high co-localization pattern with α-actinin antibody (Abcam, Cat# ab9465) that stains the Z-disk of sarcomere. (**f**) Validation of titin antibody (α-titin-CT). α-Titin-CT antibody (E-2, Santa Cruz, sc271946) recognizes the C-terminal region of titin. Immunostaining of isolated myofibrils (from mouse gastrocnemius) with α-titin-NT and α-titin-CT shows the alternating staining pattern.

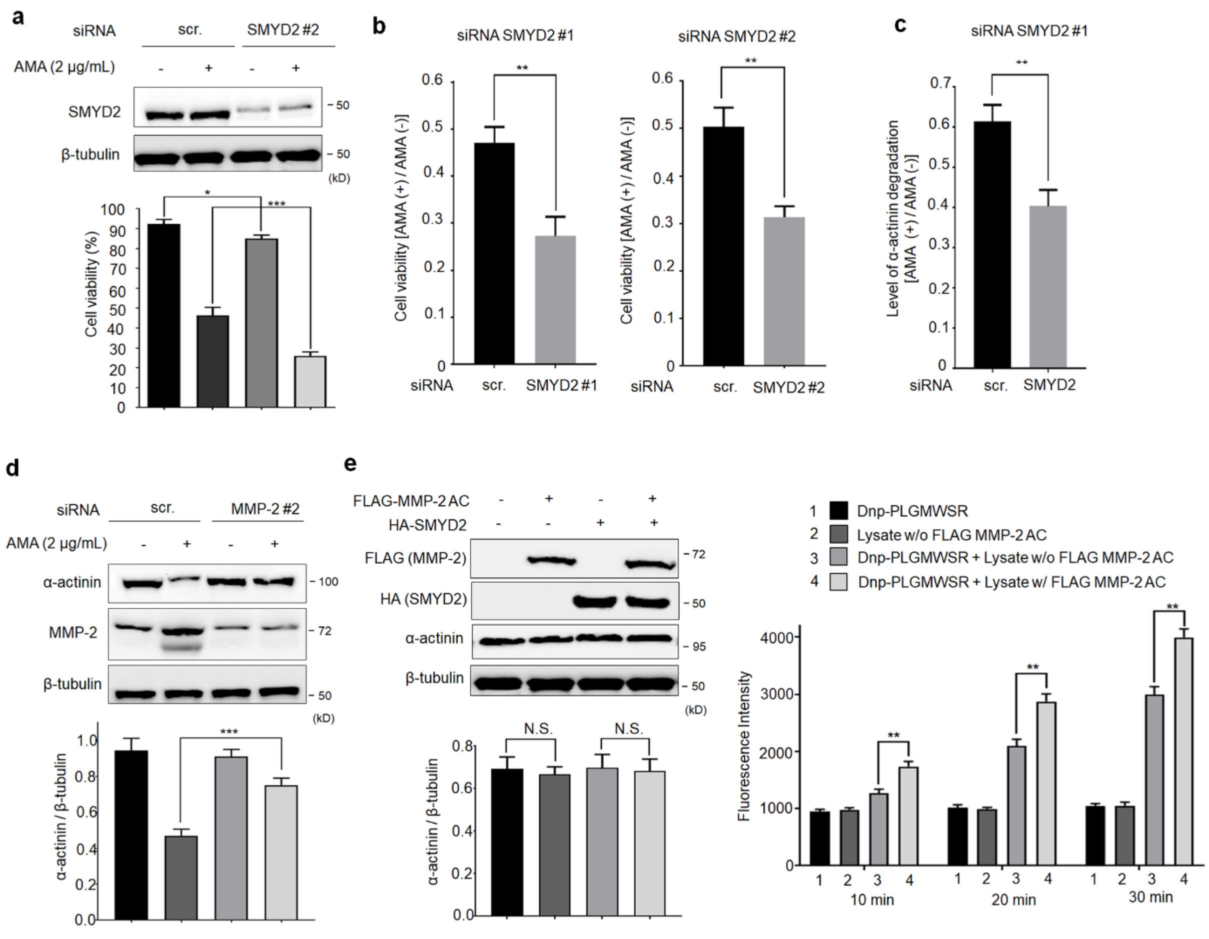


**Supplementary Figure 10. Analysis of cell viability or α-actinin degradation in cells after knockdowns of SMYD2 or MMP-2.** (**a**-**c**) Analysis of cell viability after knockdown of SMYD2. SMYD2 was knock-downed in HL-1 cells by transfecting siRNA-SMYD2-#2 (Dharmacon, Cat# M-083374-01-0005). Subsequently, AMA was incubated for 12 h. Cell viability was measured by using Trypan blue assay (**a**). The effect of SMYD2 knockdown (by two different siRNA) on cell viability (**b**) or α-actinin degradation (**c**) after normalization by values in unstressed condition (without AMA): cell viability or the level of α-actinin degradation upon treatment of AMA [AMA (+)] was divided by the respective one without AMA [AMA (-)]. Values were obtained from the celli viability in Fig. 3d and Supplementary Fig. 10a, and the level of α-actinin degradation (α-actinin / β-tubulin) in Fig. 5c. (**d**) Analysis of α-actinin degradation after knockdown of MMP-2. MMP-2 was knock-downed in HL-1 cells by transfecting siRNA-MMP-2-#2 (Dharmacon, Cat# M-093933-01-

0005). After incubation of AMA for 12 h, lysates were analyzed by Western blotting. (**e**) Overexpression of truncated active MMP-2 (FLAG-MMP-2 AC) may not be sufficient to induce degradation of α-actinin. FLAG-MMP-2 AC (amino acid 110-660) was expressed in HL-1 cells by transfection. After 24 h, lysates were analyzed by Western blotting (left). MMP-2 activity in lysates was measured by incubating a fluorogenic MMP-2 substrate (Dnp-PLGMWSR, Enzo, 0.5 mM) with lysates (right). Data represent the mean ± SD, $n = 2$ independent experiments. Difference is significant by one-way ANOVA followed by Tukey's *post-hoc* test (**a, d**) or by two-tailed Student's unpaired t-test with Welch's correction (**b**-**c**), *$p < 0.05$, **$p < 0.01$, ***$p < 0.001$, ****$p < 0.0001$.

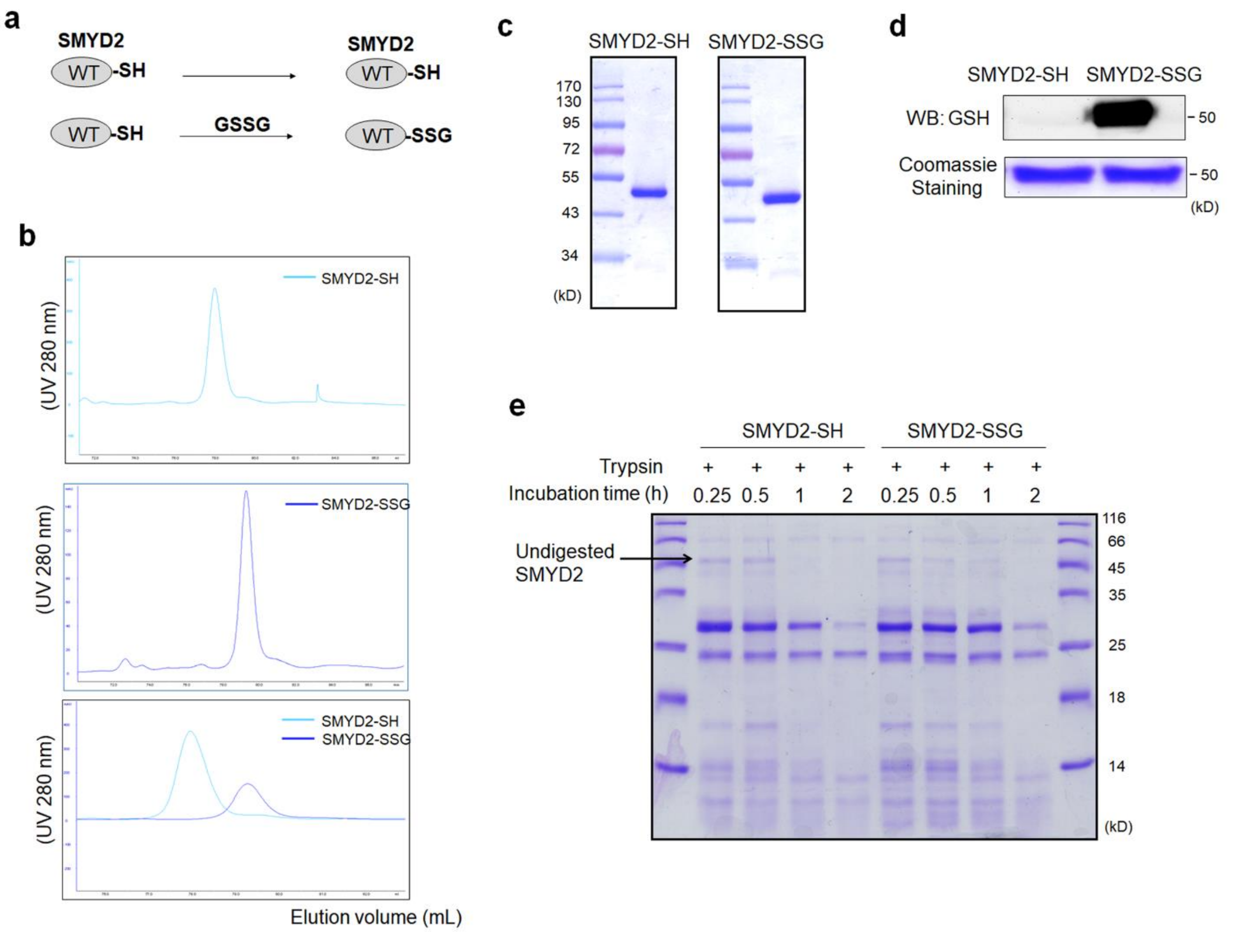


**Supplementary Figure 11. Analysis of glutathionylated SMYD2.** (**a**) A scheme for preparing non-glutathionylated SMYD2 (SMYD2-SH) and glutathionylated SMYD2 (SMYD2-SSG). (**b**) Chromatograms of ion-exchange column purification of SMYD2-SH and SMYD2-SSG. (**c**) Gel analysis after purification of SMYD2-SH and SMYD2-SSG. (**d**) Western blotting that shows glutathionylation of SMYD2. (**e**) The partial trypsin digestion of SMYD2-SH and SMYD2-SSG. Purified SMYD2-SH or SMYD2-SSG was incubated with trypsin, quenched at the indicated time, and resolved by SDS-PAGE.

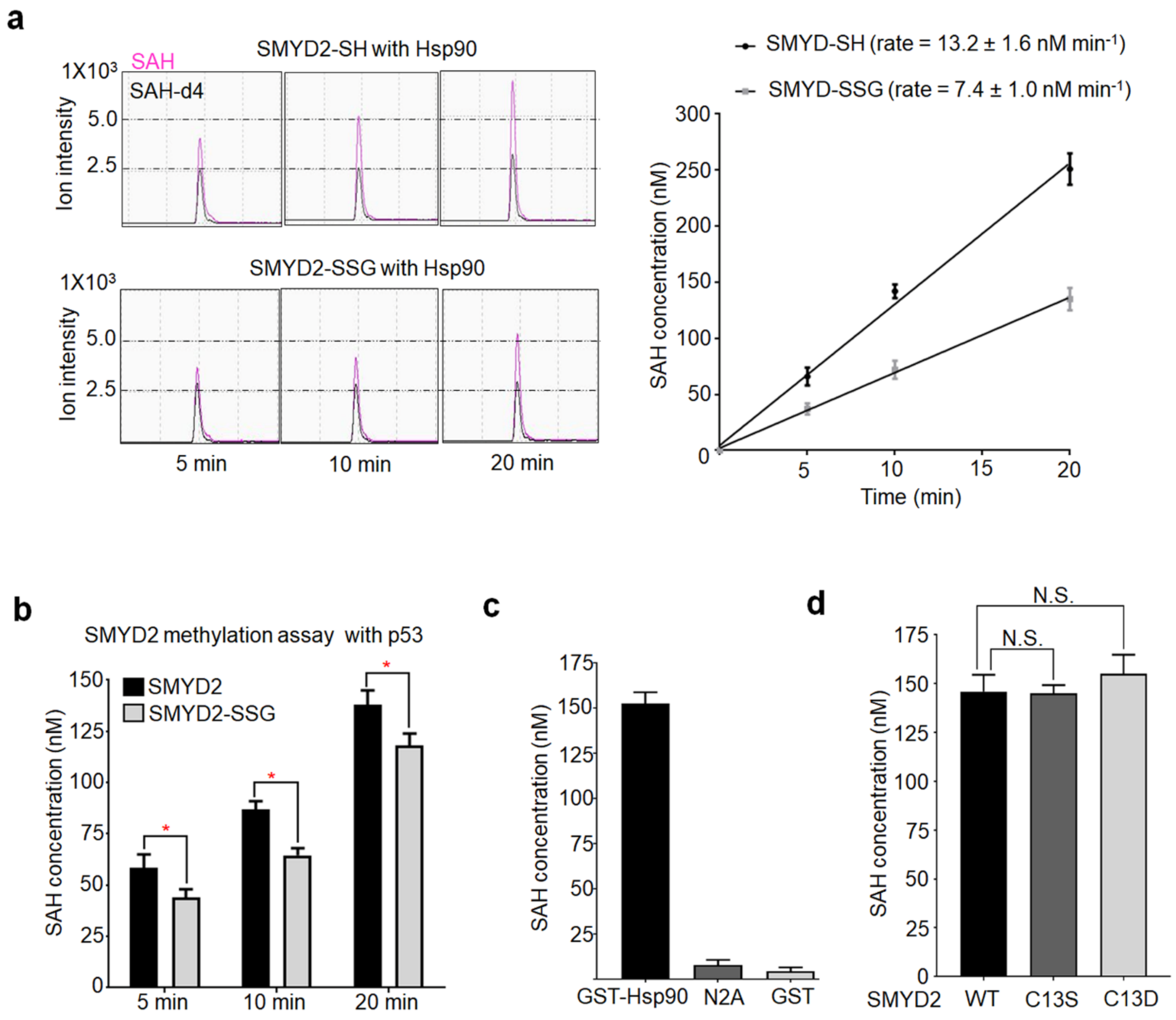


**Supplementary Figure 12. Enzyme activity of SMYD2 and glutathionylated SMYD2**. The amount of S-adenosylhomocysteine (SAH) was quantified by comparing to the amount of deuterium-labelled SAH (SAH-D4) as an internal standard in LC-MS analysis. (**a-b**) Enzyme activity of SMYD2-SH or SMYD2-SSG with Hsp90 (**a**) or 11-mer p53 peptide (HSSHLKSKKGQ) (**b**) as substrates. SMYD2-SH or SMYD2-SSG (200 nM) was mixed with Hsp90 (4 µM) or p53 peptide (25 µM) and SAM (25 µM) in TBST (pH 7.4). The SAH formation was quantified in a time-dependent manner. (**c**) Enzyme activity of SMYD2 with different substrates. SMYD2 (200 nM) was mixed with GST-Hsp90, N2A, or GST (all 4 µM) in TBST (pH 7.4). The reaction was initiated by addition of S-adenosylmethionine (SAM) (5 µM). The SAH formation was quantified after

reaction for 10 min. (**d**) Enzyme activity of SMYD2 (WT, C13S and C13D) with Hsp90 as a substrate. SMYD2 (WT, C13S and C13D) (200 nM) was mixed with Hsp90 (4 μM) and SAM (5 μM) in TBST (pH 7.4). After 10 min, SAH formation was quantified using the calibration curve. Data represent the mean ± SD, $n = 2$ independent experiments. Difference is significant by two-way ANOVA followed by Bonferroni's post-hoc test (**b**) or one-way ANOVA followed by Tukey's *post-hoc* test (**d**).

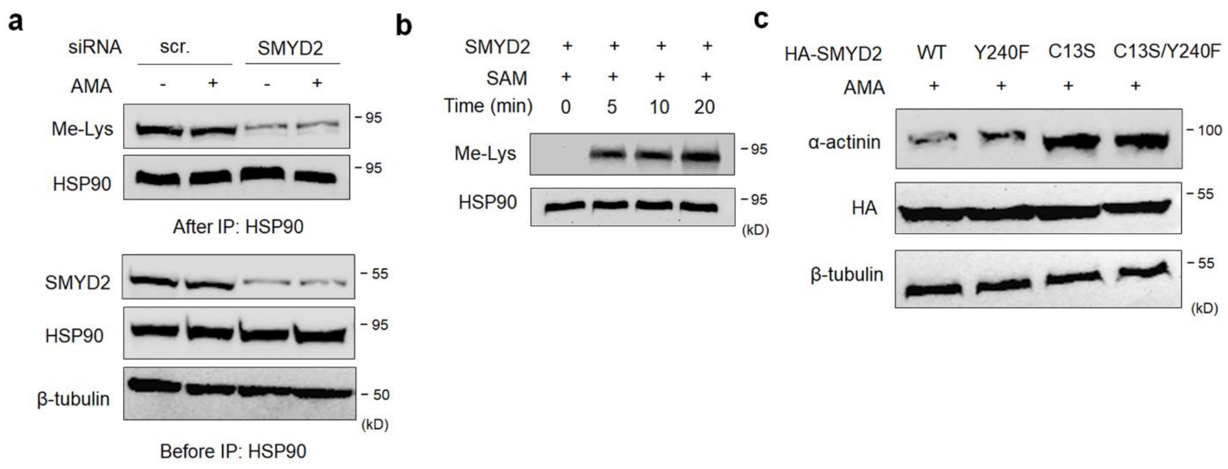


**Supplementary Figure 13. SMYD2 glutathionylation does not change methylation levels of Hsp90 in cells, and SMYD2 enzyme activity is unlikely responsible for degradation of sarcomeric proteins.** (**a**) Levels of mono-methyl lysine (Me-Lys) in Hsp90 in cells in response to AMA. After incubation of AMA to differentiated H9c2 cells for 12 h, Hsp90 was immunoprecipitated and probed for its level of Me-Lys. There is no change of methylation levels in Hsp90 upon incubation of AMA (lane 1 vs. 2), whereas SMYD2 knockdown (by using siRNA-SMYD2-#1, Santa Cruz, Cat# sc-76530) decreases Hsp90 methylation level (lane 1-2 vs. 3-4). (**b**) Valuation of Me-Lys antibody. Purified Hsp90 was methylated by incubation of SMYD2 and SAM, detecting methylation of Hsp90. (**c**) SMYD2 enzyme activity is not necessary for degradation of sarcomeric proteins. Differentiated H9c2 cells expressing SMYD2 WT or catalytically inactive mutants (Y240F) were treated with AMA for 12 h. The level of α-actinin was analyzed by Western blotting, showing no difference between enzymatically active SMYD2 and inactive SMYD2. Blots are representative of at least 3 independent experiments.

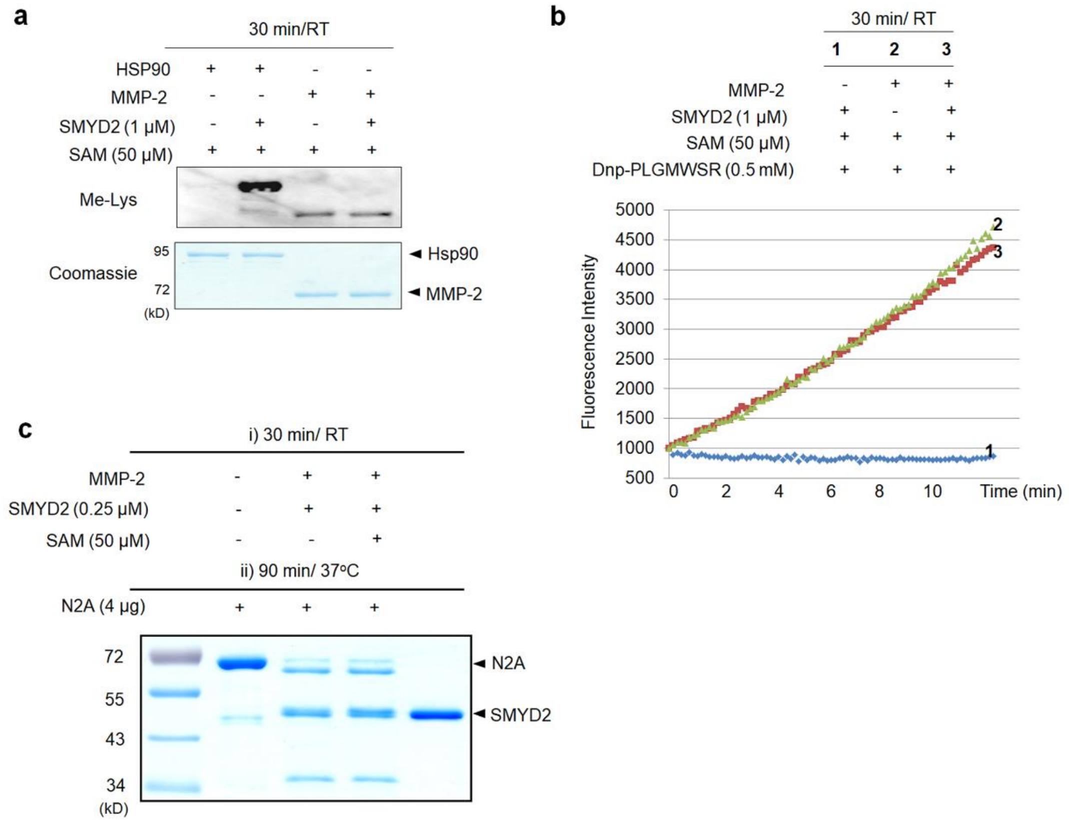


**Supplementary Figure 14. SMYD2 does not induce methylation on MMP-2 nor change the MMP-2 enzyme activity.** (**a**) Detection of mono-lysine methylation (Me-Lys) on MMP-2 by mono-lysine antibody. Purified Hsp90 or MMP-2 was incubated with SMYD2 and SAM for 30 min. The level of methylation was analyzed by mono-lysine antibody. Note the strong methylation signal of Hsp90 as opposed to no increase of methylation signal with MMP-2. (**b**) MMP-2 activity after incubation with SMYD2 and SAM. MMP-2 activity was measured by using a fluorogenic MMP-2 substrate (Dnp-PLGMWSR, Enzo) with or without pre-incubation of SAM. Note no change of MMP-2 activity after being subjected to methylation by SMYD2 and SAM. (**c**) N2A degradation by MMP-2. After pre-incubation of MMP-2 with SMYD2 and SAM for 30 min, MMP-2 was diluted to the mixture containing N2A to analyze N2A degradation. Note that the level of N2A degradation by MMP-2 subjected to the methylation condition is same as one by MMP-2 without the methylation condition. Blots are representative of at least 3 independent experiments.

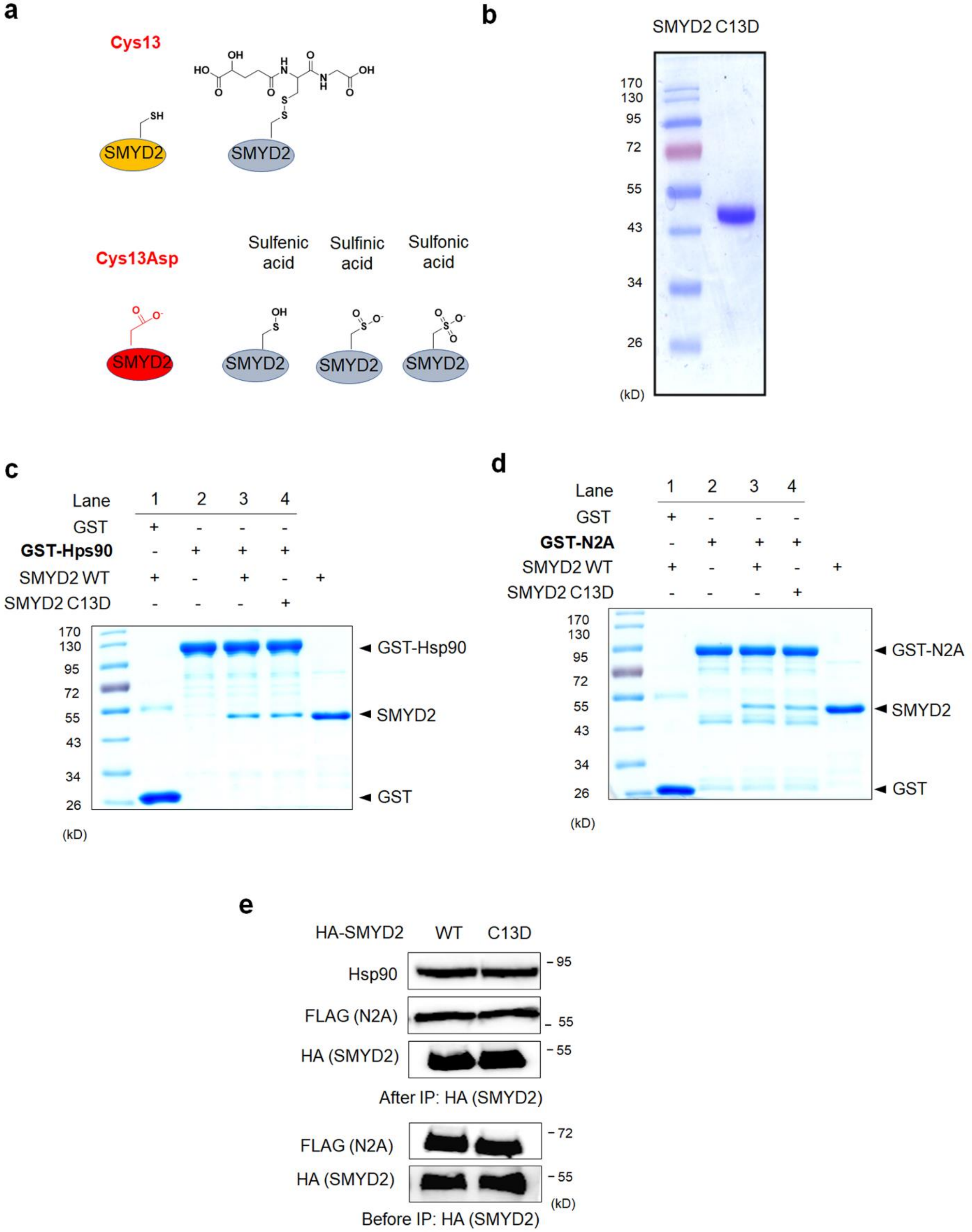


**Supplementary Figure 15.** SMYD2 C13D mutant retains the interaction with Hsp90 and N2A. (**a**) Rationale for expressing SMYD2 C13D: The size and charge of Asp are similar to ones of

oxidative cysteine modifications, such as sulfinic acid. (**b**) Gel analysis of SMYD2 C13D after purification. (**c**-**d**) Purified SMYD2 WT and C13D were incubated with GST-Hsp90 (**c**) or GST-N2A (**d**) bound to glutathione beads, and eluted samples were analyzed by Coomassie stains. (**e**) SMYD2 C13D mutant retains the interaction with Hsp90 and N2A in cells. Hsp90 or FLAG-N2A was co-immunoprecipitated with SMYD2 WT or C13D from HEK293 cells.

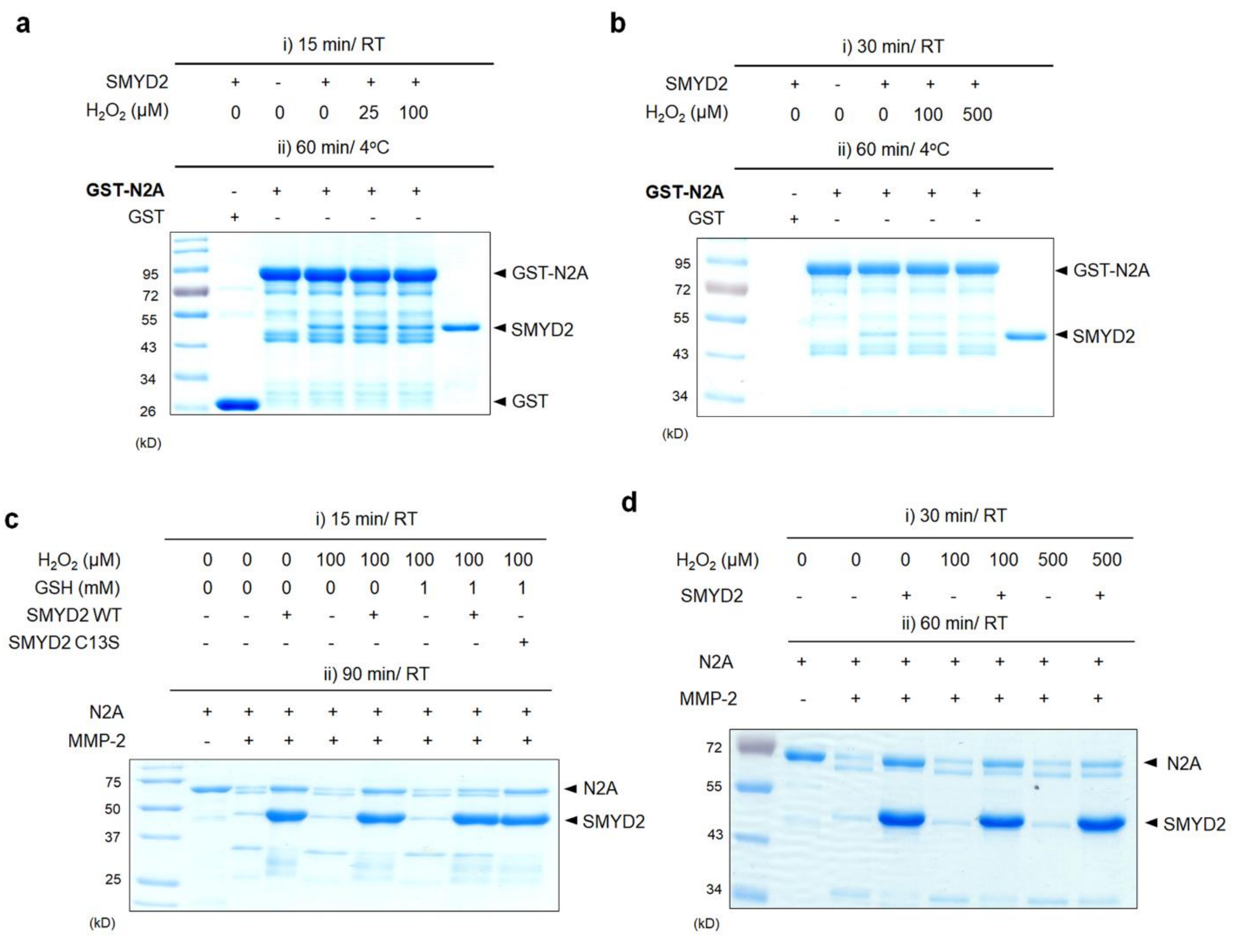


**Supplementary Figure 16. Biochemical functional analysis of SMYD2 upon oxidation by $H_2O_2$ in the presence or absence of glutathione.** (**a-b**) Analysis of the SMYD2-N2A interaction upon oxidation of SMYD2 by $H_2O_2$. SMYD2 was pre-incubated with $H_2O_2$ in low concentrations (0-100 µM) (**a**) or high concentrations (100-500 µM) (**b**) for indicated incubation times. SMYD2 was then added to the mixture (with 20-fold dilution) containing GST-N2A and glutathione-agarose. The mixture was incubated for 60 min. Bound SMYD2 was analyzed after elution from beads. (**c-d**) Analysis of MMP-2 mediated degradation of N2A after SMYD2 oxidation. SMYD2 was pre-incubated with $H_2O_2$ (0-100 µM) in the presence or absence of glutathione (1 mM) (**c**) or $H_2O_2$ (0-500 µM) (**d**). Oxidized SMYD2 was then mixed with MMP-2 and N2A for 60 min. Degradation of N2A was analyzed by running on a gel. Note a higher amount of degradation of N2A with SMYD2 that is oxidized by $H_2O_2$ (100 µM) in the presence versus absence of glutathione

in (**c**). Also, a higher amount of degradation of N2A in the presence of SMYD2 oxidized by $H_2O_2$ (500 µM) versus $H_2O_2$ (100 or 0 µM) in (**d**). Blots are representative of at least 2 independent experiments.

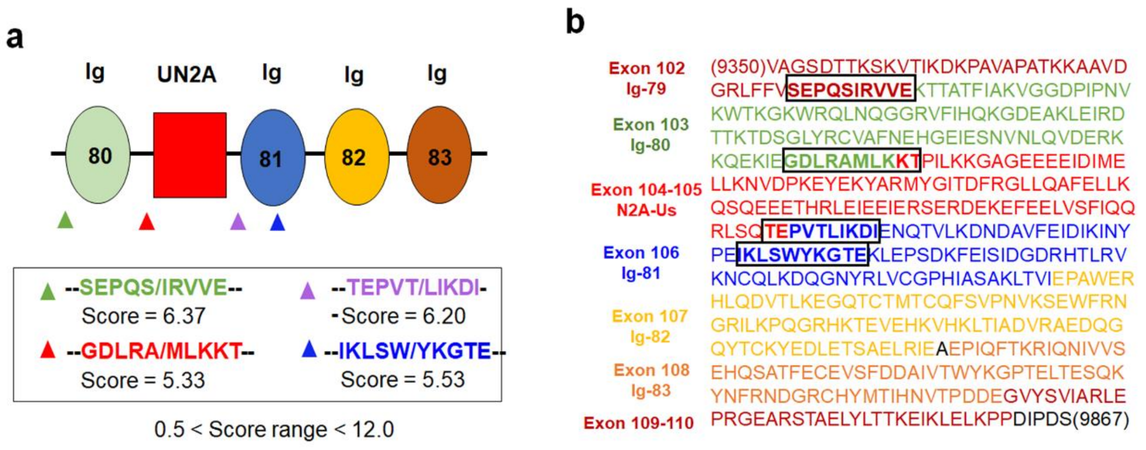


**c**

Predicted MMP2 cleavage sites in N2A domain

| | P1 | Cleaved-seq | Score | N-mass | C-mass | Fragment sizes |
|---|---|---|---|---|---|---|
| | 23 | VAPAT-KKAAV | 1.97 | 2284.25 | 57189.62 | |
| ▲ | 40 | SEPQS-IRVVE | 6.37 | 4144.23 | 55329.64 | (4 kD and 55 kD) |
| ▲ | 137 | GDLRA-MLKKT | 5.33 | 15154.93 | 44318.94 | (15 kD and 45 kD) |
| | 159 | IDIME-LLKNV | 1.62 | 17638.18 | 41835.69 | |
| | 183 | TDFRG-LLQAF | 2.22 | 20525.61 | 38948.26 | |
| | 222 | EELVS-FIQQR | 3.28 | 25294.91 | 34178.96 | |
| ▲ | 235 | TEPVT-LIKDI | 6.2 | 26822.71 | 32651.16 | (27 kD and 33 kD) |
| ▲ | 268 | IKLSW-YKGTE | 5.53 | 30720.73 | 28753.14 | (31 kD and 29 kD) |
| | 325 | IEPAW-ERHLQ | 2.9 | 37168.98 | 22304.89 | |
| | 378 | HKVHK-LTIAD | 1.25 | 43420.13 | 16053.74 | |
| | 383 | LTIAD-VRAED | 2.34 | 43933.41 | 15540.46 | |
| | 404 | ETSAE-LRIEA | 1.54 | 46349.45 | 13124.42 | |
| | 413 | AEPIQ-FTKRI | 2.91 | 47399.02 | 12074.85 | |
| | 420 | KRIQN-IVVSE | 2.5 | 48286.51 | 11187.36 | |
| | 444 | AIVTW-YKGPT | 2.21 | 50979.75 | 8494.12 | |
| | 450 | KGPTE-LTESQ | 4.91 | 51655.06 | 7818.81 | |
| | 498 | RSTAE-LYLTT | 1.44 | 57161.64 | 2312.23 | |

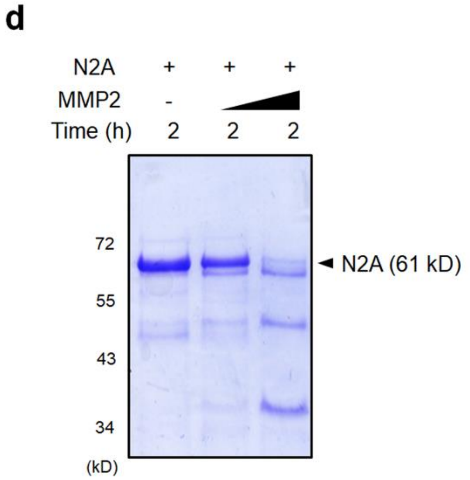


**Supplementary Figure 17.** Predicted N2A cleavage sites by MMP2 protease. The potential cleavage sites were predicted by using the MMP cleavage site prediction tool (CleavPredict). (**a**) N2A sub-domains (I80-UN2A-I81-I82-I83, Ig: Immunoglobulin domain; UN2A: a unique sequence in N2A) with potential cleavage sites that have a high score of probability. The potential cleavage sequences are shown with their positions in N2A indicated by arrow head. (**b**) The peptide sequence of N2A. N2A sub-domains are shown in different colors, and the MMP-2 cleavage sites with a high score are shown in box. (**c**) The potential MMP2 cleavage site with different scores and the resulting mass after cleavage. These are results from the MMP cleavage site prediction tool (CleavPredict). (**d**) Gel analysis of N2A degradation upon incubation of MMP-2 with an increasing amount of MMP-2. The blot is a representative of at least 3 independent experiments.

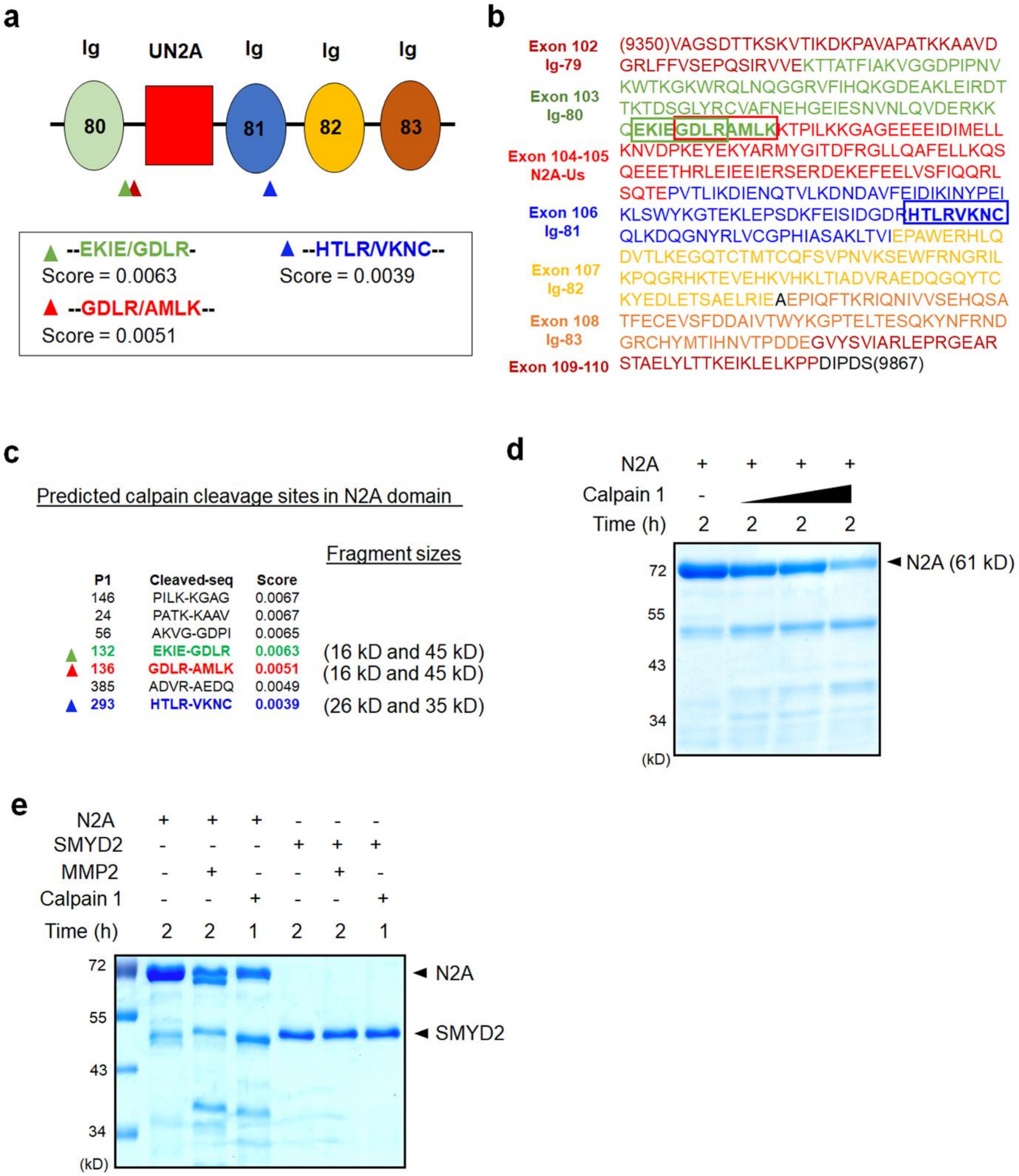


**Supplementary Figure 18.** Predicted N2A cleavage sites by calpain 1. The potential cleavage site(s) were predicted by using the calpain cleavage site prediction tool (LabCas). (**a**) N2A sub-domains (I80-UN2A-I81-I82-I83) with potential cleavage sites that have a high score of probability. The potential cleavage sequences are shown with their positions in N2A indicated by arrow head.

(**b**) The peptide sequence of N2A. N2A sub-domains are shown in different colors, and the relevant calpain 1 cleavage sites are shown in a box. (**c**) The potential calpain 1 cleavage site with different scores and the resulting mass after cleavage. (**d**) Gel analysis of N2A degradation upon incubation of calpain 1 with an increasing amount of calpain 1. (**e**) Gel analysis of N2A degradation by MMP-2 or calpain 1 in the same gel, which shows the similar size of cleaved products. SMYD2 is not degraded by MMP-2 or calpain 1. Blots are representative of at least 3 independent experiments.

## Figure 1 c

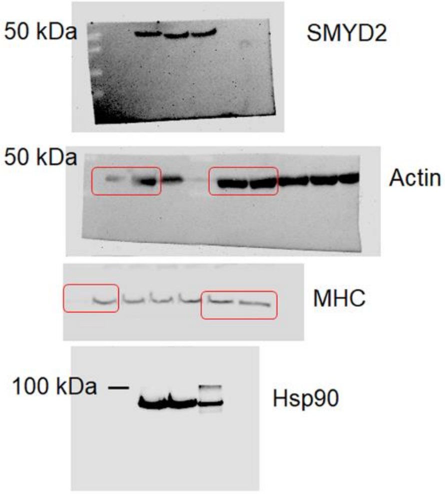


## Figure 1 d

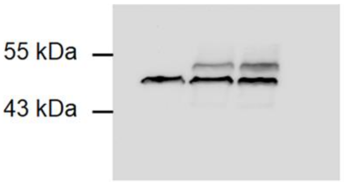


## Figure 2 h

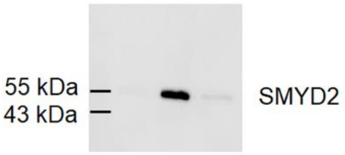


## Figure 3 a

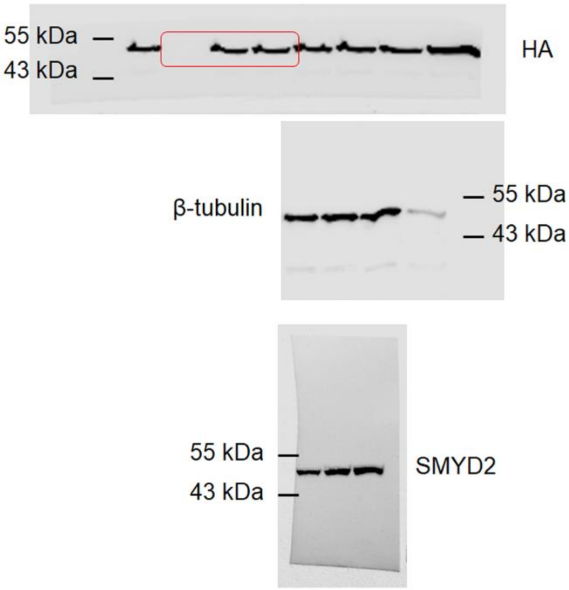


## Figure 3 d

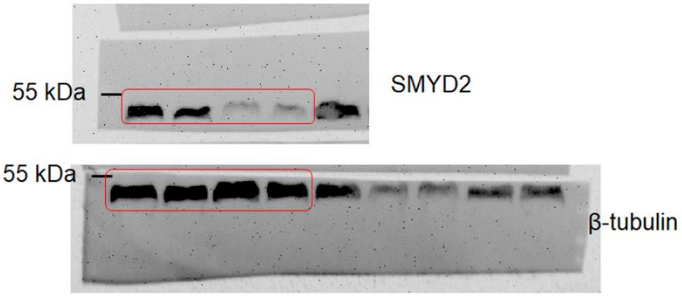

## Figure 5 a

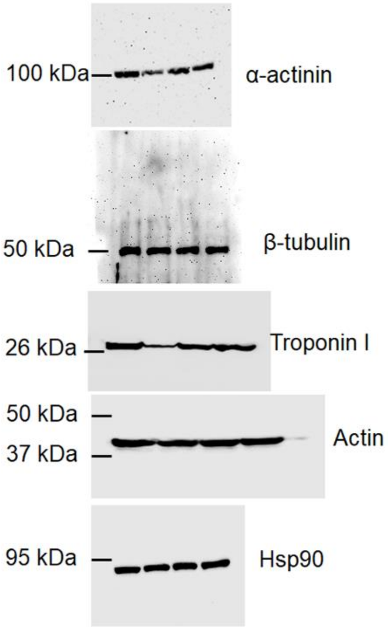


## Figure 5 c

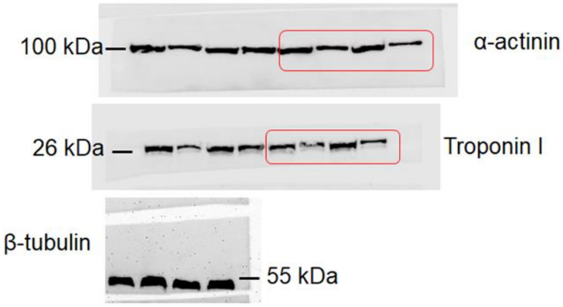


## Figure 5 d

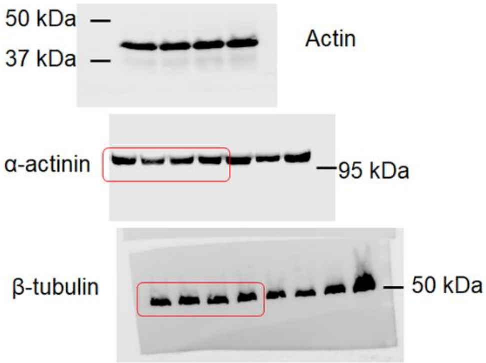


## Figure 5 e

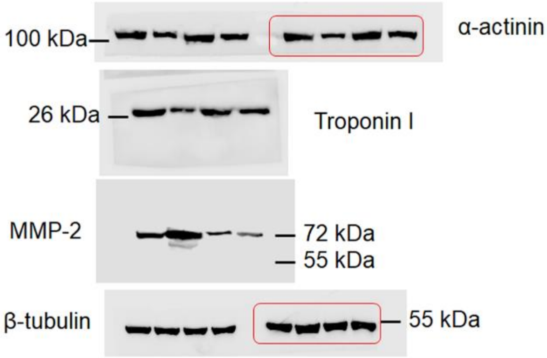


## Figure 5 g

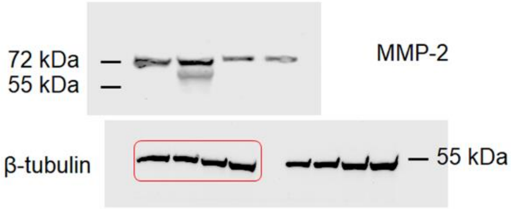

## Supplementary Figure 1 a

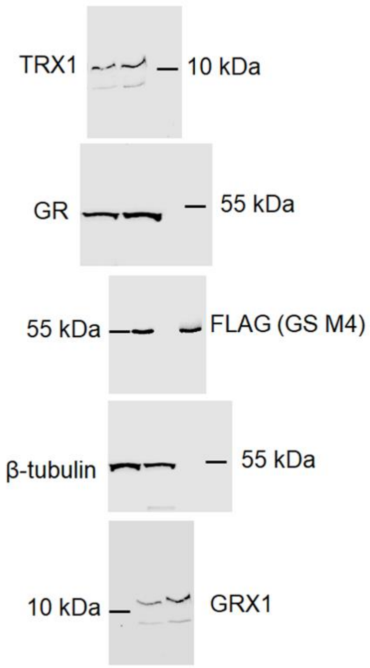


## Supplementary Figure 2 a

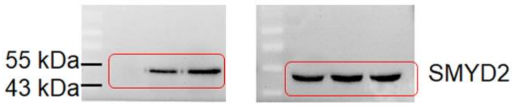


## Supplementary Figure 2 c

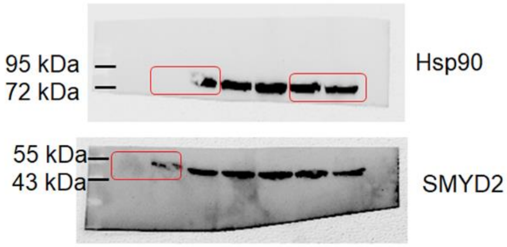


## Supplementary Figure 2 d

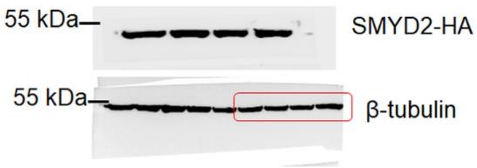


## Supplementary Figure 7 b

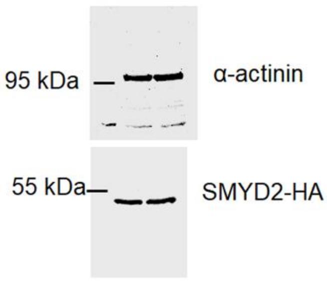


## Supplementary Figure 9 a

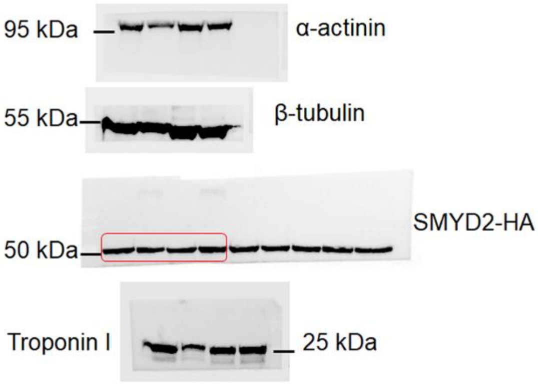


## Supplementary Figure 9 d

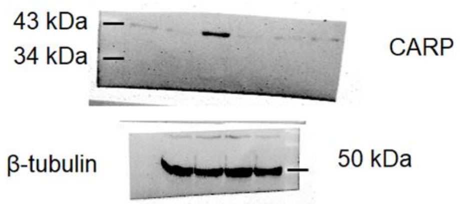

## Supplementary Figure 10 a

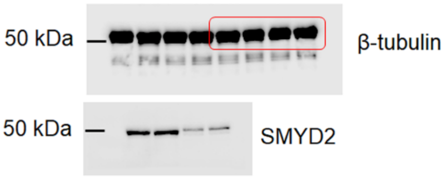


## Supplementary Figure 10 d

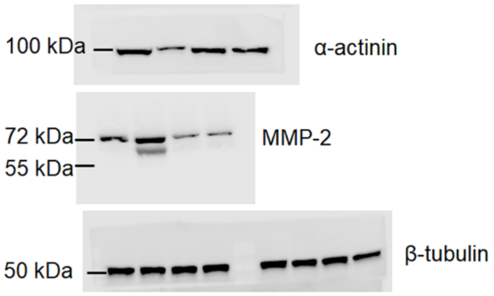


## Supplementary Figure 10 e

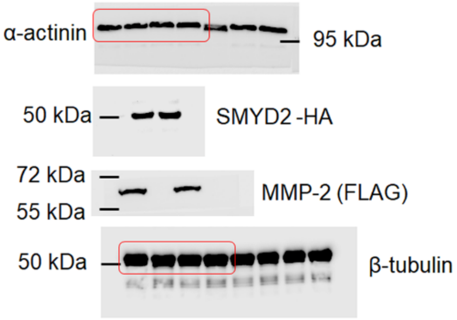


## Supplementary Figure 13 a

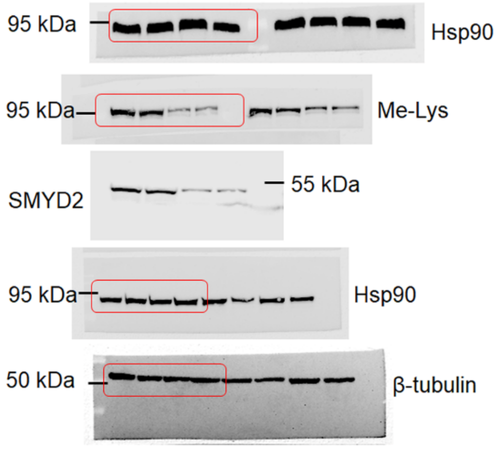


## Supplementary Figure 13 b

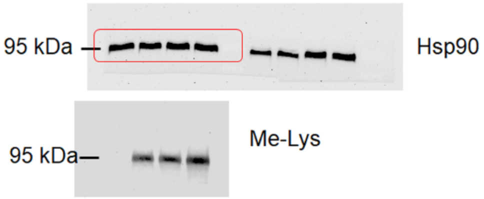


## Supplementary Figure 13 c

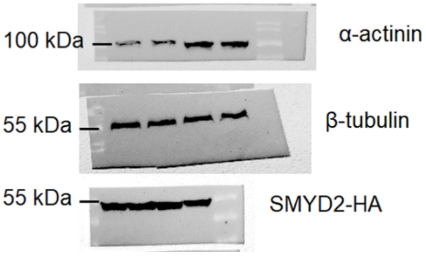


**Supplementary Figure 19.** Original blots used in Figures

## Supplementary Methods

**Materials.** All cell culture reagents, medium and Alexa-Fluor 647 goat anti-mouse IgG (H+L) secondary antibody (Cat# A21235) (Life Technologies), Amaxa Cell Line Nucleofector$^{TM}$ Kit L for H9c2 cell line (Cat# VVCA-1005 KT, Lonza), Human Heart QUICK-CLONE$^{TM}$ cDNA library (Clontech, Cat# 637213), ProLong Gold Antifade Mountant with DAPI (Cat# P36931), high capacity streptavidin-agarose beads (Cat# 20359), chemiluminescent substrates (Cat# 34080), HRV 3C Protease (Cat# PI88946), EDTA-free protease inhibitor cocktail tablets (PI88666), and Protein G Agarose beads (Cat# 15920010) (Thermo Scientific), HRP-conjugated anti-mouse (Cat# NA931), anti-rabbit secondary antibodies (Cat# NA934), and Glutathione Sepharose 4 Fast Flow beads (Cat# 17513201) (GE healthcare), Polyethyleneimine (PEI)-MAX (Cat# 247652, Polysciences, Inc), Ni-NTA agarose (Cat# 30230, QIAGEN), oxidized glutathione (Cat# 320220050, Acros Organics), S-Adenosylhomocysteine-D4 (Cayman chemicals), Human Active MMP-2 (Cat# PF023, EMD Milipore), Human Calpain 1 (Cat# C6108, Sigma), Trypsin Gold Mass Spectrometry Grade (Cat# V5280, Promega), SUMO Protease 1 (Cat# 4010, Lifesensors), ARP 100 (Cat# 2621) and acetyl-calpastatin (Cat# 2950) (Tocris Bioscience), Fluorogenic MMP-2 substrate Dnp-PLGMWSR (Cat# ALX-260-120-M005, Enzo Life Sciences), Full length MMP-2 plasmid (Cat# SC321560, Origene), EGFP-actin (pEGFP-C1 beta actin),[2] myosin heavy chain-mCherry (pCMV-mCherry-MHC-IIA)[3] and pcDNA3-EGFP mammalian vectors (Addgene).

**Cloning and mutagenesis.** PCR and cloning techniques were applied to construct the pcDNA3.1 (+)-HA-SMYD2 WT, C13S, C13D, and Y240F, pGEX-6p-2 GST-N2A, pET28a-N2A, pcDNA3.1 (+)-N2A-FLAG, pcDNA3-FLAG-SMYD2-EGFP WT and C13S, and pcDNA3.1 (+)-FLAG-MMP-2-AC (amino acids 110-660). For GST-N2A, human titin N2A domain (exon 102-109 or Ig 80-83) (UniProtKB - Q8WZ42, TITIN_HUMAN) region was amplified by PCR from human cardiac cDNA library (Clontech), and cloned into pGEX-6p2 bacterial expression vector: N2A region was

amplified by PCR with a forward primer containing EcoR1 restriction site (5’-CG CCG CGA ATT CTC ATG GTG GCT GGA AGT GAC ACT ACC AAA TCA AAA GTG ACC-3’) and a reverse primer containing Xho1 restriction site (5’- GCG CGC CTC GAG TCA GGA GTC AGG AAT ATC AGG AGG CTT CAG CTC AAG -3’), as reported previously [4]. pGEX-6p-2 plasmid and PCR product were double digested with EcoR1 and Xho1. Ligation was then performed using T4 DNA ligase. For mammalian expression vectors, human SMYD2 gene was amplified by PCR with a forward primer containing Kozak sequence and Nhe1 restriction site (5’-GCG CGC GCT AGC ACC ATG AGG GCC GAG GGC CTC -3’) and a reverse primer containing C-terminal HA-tag and Xho1 restriction site (5’-GCG CGC CTC GAG TCA GGC ATA GTC GGG CAC GTC ATA CGG ATA GTG GCT TTC AAT TTC CTG TTT GAT CTC AG-3’). N2A domain was amplified by PCR with a forward primer containing Kozak sequence, N-terminal FLAG-tag, and BamH1 restriction site (5’-GCC GCC GGA TCC ACC ATG GAC TAC AAA GAC GAT GAC GAC AAG GTG GCT GGA AGT GAC AC-3’) and a reverse primer containing Xho1 restriction site (5’-CGC CGC CTC GAG TCA GGA GTC AGG AAT ATC AGG AG-3’). A truncated MMP-2 region (amino acids 110-660) was amplified by PCR using a full length MMP-2 plasmid (Origene, SC321560) with a forward primer containing Kozak sequence and HindIII restriction site (5’- GCA AGC AAG CTT ACC ATG GCC AAC TAC AAC TTC TTC CCT CG -3’) and a reverse primer containing C-terminal FLAG and Xho1 restriction site (5’-GCA AGC CTC GAG TCA CTT GTC GTC ATC GTC TTT GTA GTC GCA GCC TAG CCA GTC GGA TTT G-3’). All PCR products were sub-cloned into pcDNA3.1/hygro (+) mammalian vector by using the corresponding restriction enzymes. For pcDNA3-FLAG-SMYD2-EGFP, human SMYD2 gene was amplified by PCR with a forward primer containing Kozak sequence and BamH1 restriction site (5’- GCA AGC GGA TCC ACC ATG AGG GCC GAG GGC CTC -3’) and a reverse primer with Xho1 restriction site (5’- GCA AGC CTC GAG GTG GCT TTC AAT TTC CTG TTT GAT CTC AG -3’). The PCR product was sub-cloned into pcDNA3-EGFP mammalian vector (Addgene, 13031) by using BamH1 and Xho1 sites. All bacterial and mammalian mutant vectors of SMYD2 were constructed by quick change

mutagenesis with following primers: SMYD2 C13S (forward: 5'-GGC CTG GAG CGC TTC AGC AGC CCG GGC AAA GGC-3' and reverse: 5'-GCC TTT GCC CGG GCT GCT GAA GCG CTC CAG GCC -3'), C13D (forward: 5'-GGC CTG GAG CGC TTC GAC AGC CCG GGC AAA GGC-3' and reverse: 5'-GCC TTT GCC CGG GCT GTC GAA GCG CTC CAG GCC-3'), Y240F (forward: 5'-G GAG GTT TTT ACC AGC TTT ATT GAT CTC CTG TAC CC-3' and reverse: 5'-GG GTA CAG GAG ATC AAT AAA GCT GGT AAA AAC CTC C-3'). The entire open reading frame (ORF) of all prepared plasmids was confirmed by DNA sequencing.

**Bacterial expression and purification of SMYD2 WT and mutants.** BL21 (DE3) cells were transformed with pCDF-SUMO-SMYD2 (WT, C13S, C13D, or Y240F). Transformants were grown in LB medium (2 L) at 37°C until $OD_{600}$ reached about 0.5. The protein expression was induced with IPTG (0.1 mM) and incubated overnight at 16°C. Cells were harvested by centrifugation at 8,500 x g for 20 min and lysed by passing French Press three times. Cell lysate was centrifuged at 35,200 x g for 30 min, and the soluble fraction was subjected to $Ni^{2+}$-NTA affinity column. The eluted protein was incubated with SUMO Protease 1 at 4°C for 10 h in a buffer (50 mM Tris-HCl pH 7.4, 150 mM NaCl and 6 mM β-mercaptoethanol). The mixture was then incubated with $Ni^{2+}$-NTA beads to remove His-SUMO tag and His-SUMO protease. Cleaved SMYD2 was collected in the flow-through. SMYD2 was purified by anion exchange column chromatography using Mono Q$^{TM}$ 5/50 GL cation column (GE Healthcare) with buffer A (50 mM Tris-HCl pH 8.0) and buffer B (50 mM Tris-HCl, pH 8.0, and 1 M NaCl) to homogeneity and concentrated to 4-5 mg/ml. The purified protein was stored in a buffer (50 mM Tris–HCl pH 8.0, 150 mM NaCl, 0.2 mM β-mercaptoethanol and 5% glycerol). The same approach was applied to express the GST-Hsp90 and GST-N2A where proteins were incubated with glutathione beads for purification, and proteins were stored in a buffer (50 mM Tris–HCl pH 8.0, 150 mM NaCl, 0.2 mM β-mercaptoethanol and 5% glycerol). GST on N2A domain was cleaved by HRV 3C Protease. Briefly, GST-N2A was incubated with the HRV 3C protease (enzyme/substrate = 1/50) overnight at 4°C in the supplied

HRV 3C protease reaction buffer. The cleaved mixture was incubated with pre-washed glutathione beads for 2 h at 4°C. The cleaved N2A domain was then collected in the flow-through and stored in the same buffer (50 mM Tris–HCl pH 8.0, 150 mM NaCl, 0.2 mM β-mercaptoethanol and 5% glycerol).

**Preparation of glutathionylated SMYD2 *in vitro*.** SMYD2 WT protein was incubated in a buffer (100 mM Tris-HCl pH 7.4 and 150 mM NaCl) with and without oxidized glutathione (GSSG) (1 mM) at 4°C for 4 h. Proteins were then purified by anion exchange column chromatography using Mono Q$^{TM}$ 5/50 GL cation column (GE healthcare). A linear gradient between buffer A (50 mM Tris-HCl, pH 8.0) and buffer B (50 mM Tris-HCl, pH 8.0 and 1 M NaCl) was used while monitoring absorbance at 280 nm. Purified proteins were dialyzed in a buffer (50 mM Tris–HCl pH 8.0, 150 mM NaCl and 10% glycerol), concentrated, flash-frozen in liquid-nitrogen, and stored at -80$^{o}$C. Protein concentration was determined by Bradford assay (BioRad).

**Partial trypsin digestion of SMYD2.** The same amount of purified SMYD2-SH and SMYD2-SSG (5 µg) were mixed with trypsin (1 µg) at 37°C. Digestion was quenched in different time points (15, 30, 60 and 120 min) by addition of an SDS-loading buffer. Digested samples were separated by SDS-PAGE and visualized by Coomassie stains.

***In vitro* SMYD2 methyltransferase activity assay.** SMYD2 methyltransferase activity was determined by measuring the amount of S-adenosylhomocysteine (SAH) generated from the enzymatic reaction. SAH was quantified by the multiple reactions monitoring (MRM) mode in LC-MS/MS with SAH-d4 as an internal standard. In MRM mode, the mass transition from the precursor to product was used to detect SAH (*m/z* 385 to 136) and SAH-d4 (*m/z* 389 to 136) with peak quantification.[5] The UV absorbance at 260 nm and a molar extinction coefficient[6] of 15,400 $M^{-1}cm^{-1}$ were used to determine the concentartion of SAM and SAH. The calibration curve was

made by plotting the integrated peak sizes versus SAH concentrations (10, 50, 100, 250 and 1000 nM). The activity assay was carried out at room temperature in a buffer (25 mM Tris-HCl, pH 8.0, and 0.01% Tween 20). SMYD2 (200 nM) was incubated with individual substrates, including p53 (25 μM), Hsp90 (4 μM), N2A (4 μM), Hsp90-GST (4 μM), and GST (4 μM), followed by addition of SAM (5 or 25 μM) at room temperature. Methylation was quenched at different time points by addition of TCA (5%).

**Detection of Hsp90 methylation.** For detection of Hsp90 methylation, differentiated H9c2 cells without or with SMYD2 knockdown were treated with AMA (2 μg/mL). After lysis, Hsp90 was immunoprecipitated by using mouse Hsp90 antibody (BD transduction, Cat# 610418) and probed with rabbit mono-methyl lysine antibody (me-K) (Cell Signaling, Cat#14679) (1:1000). Similarly, purified Hsp90 or MMP-2 was incubated with SMYD2 and SAM at room temperature. Then reaction was quenched by addition of an SDS-loading buffer. Methylation of Hsp90 or MMP2 was probed by Western blotting with me-K antibody.

**Detection of SMYD2 oxidation (sulfonic acid).** Differentiated H9c2 cells expressing SMYD2-HA WT or C13S was treated with AMA (2 μg/mL) for 12 h. Cells were then lysed with a lysis buffer containing iodoacetamide (25 mM). HA-SMYD2 was immunoprecipitated by mouse HA-antibody (Biolegend, Cat# 901502). The blot was probed with rabbit cysteine (sulfonate) antibody (Enzo Life Sciences, Cat# ADI-OSA-820-D, 1:1000), which can detect sulfinic acid or sulfonic acid. The rabbit cysteine (sulfonate) antibody was validated with purified SMYD2 *in vitro*. Briefly, SMYD2 was incubated with $H_2O_2$ (10 mM) for 20 min at room temperature or after pretreatment of iodoacetamide (25 mM) to block cysteine oxidation as a negative control. SMYD2 was analyzed by Western blotting with Hsp90 antibody or cysteine (sulfonate) antibody.

**Measuring MMP2 activity.** A peptide-based fluorogenic substrate (Dnp-PLGMWSR, Enzo Life Sciences) was used to examine MMP-2 activity by measuring fluorescence (ex: 280 nm and em: 360 nm) at different time intervals. To examine MMP-2 activity after being subjected to the SMYD2 methylation condition, MMP-2 (0.05 µg) was subjected to methylation in a buffer containing SMYD2 (1 µM) and SAM (50 µM) for 30 min at room temperature, which was then added to the MMP-2 digestion buffer containing a fluorogenic substrate (0.5 mM). The fluorescence was monitored over 15 min period at 37°C. To measure MMP-2 activity after ectopic expression of FLAG-MMP-2 AC, cell lysates (25 µg) with and without overexpression of FLAG-MMP-2 AC were diluted into the MMP-2 digestion buffer containing a fluorogenic substrate (0.5 mM). The fluorescence was monitored for 30 min at 37°C.

**Confocal microscope setting**. Confocal images were captured by Zeiss LSM 780 confocal microscope with the following setting: image dimensions (x: 1024, y: 1024), image bit depth (8 bit), scan mode (plane), pixel dwell (1.58 µs), objectives (Plan-Apochromat 63x/1.40 Oil DIC M27 and EC Plan-Neofluar 40x/1.30 Oil DIC M27)

**FACS analysis.** Transfection efficiency of SMYD2 WT and C13S was measured by FACS analysis. H9c2 myoblast were transfected with pcDNA3-SMYD2-EGFP WT or C13S by electroporation and maintained in differentiation medium. After differentiation, cells were collected by addition of trypsin. Transfection efficiency was analyzed by using BD LSR II Flow Cytometer and data were processed by FlowJo software.

**Dot-blot analysis of titin.** Differentiated H9c2 or HL-1 cells expressing SMYD2-HA WT or C13S were subjected to serum starvation for 12 h and treated with AMA (2 µg/mL). Titin was immunoprecipitated.[7, 8] First, cells on a plate were washed twice with PBS containing $MgCl_2$ (2 mM) and EGTA (1 mM), and incubated with an extraction buffer (100 mM KCl, 10 mM Pipes, pH

6.8, 300 mM sucrose, 2 mM $MgCl_2$, 1 mM EGTA, 0.1 mM PMSF, 10 μM E-64, 100 μM leupeptin, protease inhibitor cleavage cocktail and DNase1) containing 0.5% Triton X-100 for 10 min on ice. After collecting the initial extractable fraction, Triton-resistant material was scraped into the cytoskeletal-fraction-removal buffer (the extraction buffer with 2% SDS, 75 mM β-mercaptoethanol and 100 mM NaCl, instead of KCl). The scraped portions were then heated at 60°C for 10 min and passed through a syringe (1 mL size) equipped with a needle for 5-10 times. Lysates were diluted with a non-denaturing lysis buffer (20 mM Tris HCl pH 8, 140 mM NaCl, 1% Triton X-100 and 2 mM EDTA) and dialyzed against the same non-denaturing lysis buffer containing 0.05% SDS and protease inhibitors. Cell lysates (5 mg) were mixed with titin (E-2) mouse antibody (α-titin-CT) (Santa Cruz, sc271946) for 4 h at 4$^{o}$C. This mixture was then incubated with Protein-G agarose beads (pre-washed twice with a non-denaturing buffer) overnight at 4$^{o}$C. Beads were washed three times with a non-denaturing buffer. Bound proteins were eluted using a 0.05% SDS buffer by boiling beads at 90°C for 10 min. Eluted proteins were then diluted and blotted onto PVDF membrane, using Bio-Rad Bio-Dot Blot micro-filtration apparatus with 96 wells. Membrane was blocked by a blocking buffer (1xTBS with 0.1% TWEEN-20 and 3% BSA) for 1 h at room temperature, and probed with the following primary antibodies: titin rabbit antibody (Novus biologicals, Cat# NBP 1-88071)(1:1000) (α-titin-NT), titin mouse antibody (Santa Cruz, sc271946) (α-titin-CT) (1:1000), HA-antibody (Biolegend, Cat# 901502)(1:1000), and β-tubulin (Santa Cruz) (1:1000).

### Supplementary References


1. Morrill, E.E. *et al.* A validated software application to measure fiber organization in soft tissue. *Biomech. Model Mechanbiol.* **15**, 1467-1478 (2016).
2. Watanabe, N. & Mitchison, T.J. Single-molecule speckle analysis of Aactin filament turnover in lamellipodia. *Science* **295**, 1083-1086 (2002).
3. Dulyaninova, N.G., House, R.P., Betapudi, V. & Bresnick, A.R. Myosin-IIA heavy-chain phosphorylation regulates the motility of MDA-MB-231 carcinoma cells. *Mol. Biol. Cell.* **18**, 3144-3155 (2007).

4. Voelkel, T. *et al.* Lysine methyltransferase Smyd2 regulates Hsp90-mediated protection of the sarcomeric titin springs and cardiac function. *Biochim. Biophys. Acta* **1833**, 812-822 (2013).
5. Wang, L. *et al.* Structure of Human SMYD2 Protein Reveals the Basis of p53 Tumor Suppressor Methylation. *J. Biol. Chem.* **286**, 38725-38737 (2011).
6. Krijt, J., Duta, A. & Kozich, V. Determination of S-Adenosylmethionine and S-Adenosylhomocysteine by LC-MS/MS and evaluation of their stability in mice tissues. *J. Chromatogr. B* **877**, 2061-2066 (2009).
7. Isaacs, W.B., Kim, I.S., Struve, A. & Fulton, A.B. Biosynthesis of Titin in Cultured Skeletal-Muscle Cells. *J. Cell. Biol.* **109**, 2189-2195 (1989).
8. Isaacs, W.B., Kim, I.S., Struve, A. & Fulton, A.B. Association of Titin and Myosin Heavy-Chain in Developing Skeletal-Muscle. *Proc. Natl. Acad. Sci. USA* **89**, 7496-7500 (1992).
